# Topological analysis of sharp-wave ripple waveforms reveals input mechanisms behind feature variations

**DOI:** 10.1038/s41593-023-01471-9

**Published:** 2023-11-09

**Authors:** Enrique R. Sebastian, Juan P. Quintanilla, Alberto Sánchez-Aguilera, Julio Esparza, Elena Cid, Liset M. de la Prida

**Affiliations:** 1 Instituto Cajal. CSIC, Madrid, Spain; 2 Present Address: Department of Physiology, Faculty of Medicine, Universidad Complutense de Madrid, Madrid, Spain

**Keywords:** Neural circuits, Hippocampus, Neurophysiology

## Abstract

The reactivation of experience-based neural activity patterns in the hippocampus is crucial for learning and memory. These reactivation patterns and their associated sharp-wave ripples (SWRs) are highly variable. However, this variability is missed by commonly used spectral methods. Here, we use topological and dimensionality reduction techniques to analyze the waveform of ripples recorded at the pyramidal layer of CA1. We show that SWR waveforms distribute along a continuum in a low-dimensional space, which conveys information about the underlying layer-specific synaptic inputs. A decoder trained in this space successfully links individual ripples with their expected sinks and sources, demonstrating how physiological mechanisms shape SWR variability. Furthermore, we found that SWR waveforms segregated differently during wakefulness and sleep before and after a series of cognitive tasks, with striking effects of novelty and learning. Our results thus highlight how the topological analysis of ripple waveforms enables a deeper physiological understanding of SWRs.

## Abstract (web summary)

This study applies topological analysis to hippocampal ripple waveforms, uncovering a low-dimensional continuum that encodes layer-specific synaptic input information. It also reveals how ripple waveforms vary during wakefulness, sleep and learning.

## Main

Cognitive processes essential for adaptive behavior, such as navigation and memory, rely on hippocampal activity. SWRs are local field potential (LFP) events underlying memory recall and consolidation^1^. They have been reported in both mammals (rodents, monkeys and humans) and non-mammals (birds and reptiles), spanning an oscillatory range from 80 Hz to 250 Hz (ref. ^2^). During SWRs, neurons fire in sequences representing experience reactivated in the forward and reverse order^3–5^, and single-cell studies have reported cell-type-specific firing patterns^6–9^. As SWRs interact in a brain-wide manner, intra-hippocampal and extra-hippocampal inputs act to shape their features^10–12^. Their organization is influenced by factors such as novelty, learning and experience^13–15^, but identifying the direction of variations is not trivial. While LFP signals are known to encode cognitively relevant information^16,17^, analysis of SWRs mostly relies on estimating their mean spectral characteristics, posing limits to our understanding of these events.

**Fig. 1:**
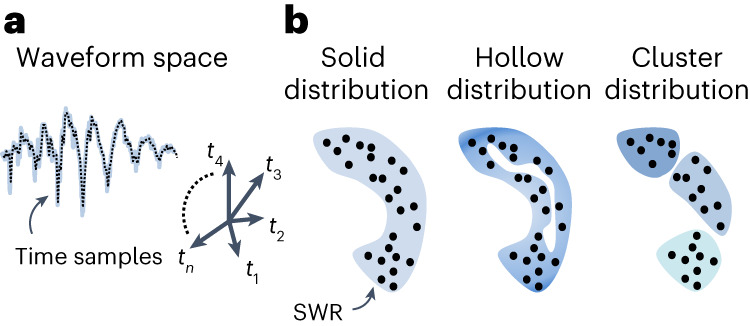
The problem of SWR classification based on ripple waveforms. **a**, Ripples are analyzed in the waveform space, which is defined from time samples in a given window. **b**, Each event becomes a point in the cloud made from all SWRs. The cloud could adopt different shapes from continuous (solid or hollow) to clustered. Topological data analysis of SWR events in the waveform space will help to disambiguate between the different distributions.

More recently, using unsupervised methods, it has been suggested that SWR waveforms can carry much more information than can be inferred from spectral approaches^7,10,18^. An open question is whether SWRs can be classified in a finite number of categories, or whether they just reflect a continuum of waveforms that can be characterized according to their features (for example, slope, amplitude and frequency). Previous attempts have used different methods, from spectral decomposition to unsupervised analysis of SWRs in a predefined feature space, reaching different conclusions^7,10,18–20^. Importantly, when dealing with methods that implicitly look for clusters and/or rely on principal feature distributions, results could be misleading. To fill this gap, we transformed SWR classification into an unbiased topological problem by projecting LFP ripple traces into a high-dimensional waveform space (Fig. 1a). Here, the dimension of the waveform space is determined by the sampling rate of SWRs. Events of similar waveforms will lie close together, while those of different characteristics will be separated. We then apply methods from topological data analysis to characterize the shape of the SWR cloud using persistent homology^21^, which inform us about the distribution of points in the data cloud (Fig. 1b). By directly estimating the intrinsic dimension^22^ in the original waveform space, dimensionality reduction techniques can then be applied for visualization and quantification using structural indices^23^. These topological methods enable unbiased data-driven approaches to identify the sources of variability of SWR waveforms.

Adopting this approach allowed us to address some unresolved questions in the field. Do SWRs form a continuum of events, or do they rather segregate into different categories? Can unsupervised analysis of ripple waveforms provide relevant mechanistic information about a diversity of SWRs? Are SWRs emitted during the awake and the sleep states that follow learning differently influenced by cognitive demands? We show that an unbiased topological characterization of ripple waveforms provides physiologically relevant information that cannot be recovered from a simple feature space.

## Results

### Topological analysis of ripple waveforms

**Fig. 2:**
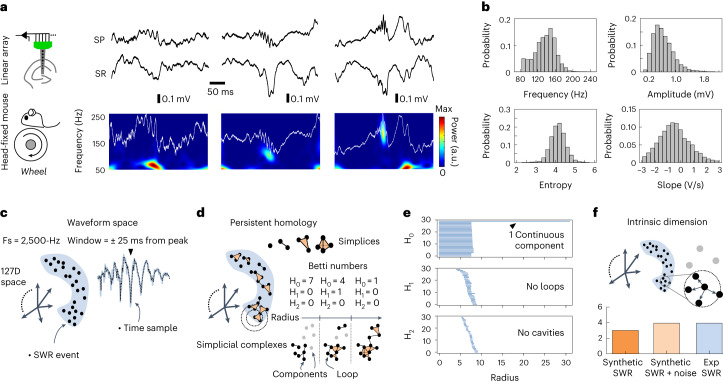
Topological analysis of SWR waveforms. **a**, Experimental setup and examples of SWRs. Note event-to-event variability in terms of LFP waveforms and spectra from low (80 Hz, leftmost) to high frequencies (>160 Hz, rightmost). **b**, Distribution of SWR features (frequency, amplitude, spectral entropy and slope) from all SWR events (10,741 events, 58 independent sessions, 27 mice). **c**, Ripples recorded at the SP are represented in a high-dimensional waveform space (127D) determined by the sampling rate (2,500 Hz) and the window size (±25 ms). In this representation, each SWR is a point in the cloud made by all detected events. **d**, Topology of the SWR cloud was examined with persistent homology, which identifies holes and cavities in the data by using simplicial complexes. These are made of simplices (for example, dots, lines, triangles and tetrahedrons) that connect data points (SWRs in this case) in a given radius. Persistent homology looks for their persistence as the radius around each point varies in the high-dimensional space. The different homology groups (H) are defined from the number of cuts that separate simplices in pieces. **e**, Barcodes for the three homology groups (H_o_, H_1_ and H_2_) show persistence of only one continuous component in the experimental SWR data cloud (data from a random subset of *n* = 3,500 events). **f**, The intrinsic dimension of the SWR data cloud was estimated using the ABID method^22^, which looks at the distribution of angles among the neighbors of a data point in a given radius. Bars represent single values.

SWRs were recorded from the dorsal CA1 stratum pyramidale (SP) and stratum radiatum (SR) of awake head-fixed mice using linear arrays (Fig. 2a). Events were detected and visually validated following consensus methods reported by us and others^2^ (Methods). SWRs exhibited variability in terms of frequency, amplitude, spectral entropy and slope among other features typically used for their characterization^2,7^ (Fig. 2b). For instance, SWRs of low (80–100 Hz) and high (>160 Hz) dominant frequencies intermingled with different amplitudes and slopes in a given recording session (Fig. 2a).

**Extended Data Fig. 1:**
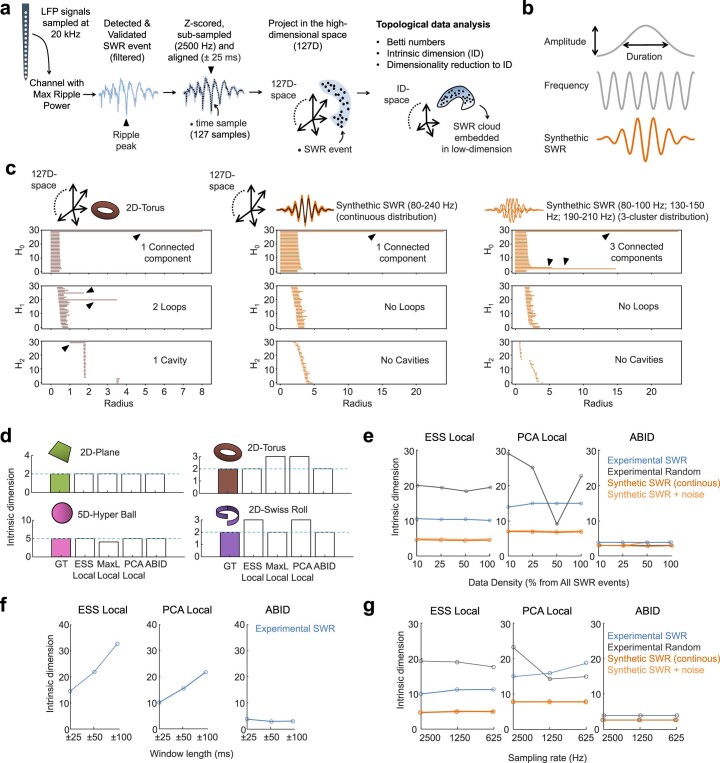
Topological analysis of SWR waveforms in the high-dimensional space. **a**, Flow diagram of the method. LFP signals from the channel with the maximal ripple power are selected (note that this is not limited to silicon probe recordings). First, the timing of all detected and validated SWR are used to center analysis. Next, LFP signals are subsampled (for example 2500 Hz; see other sampling rates in g) and all validated events aligned around the ripple peak (for example ±25 ms; see other window lengths in f). For a given sampling rate and window length there is a fixed number of time points making the SWR (for example 127 points considering the first and latest samples). The third step is to project each SWR event into the high-dimensional space defined by temporal samples (for example 127D). All events will form a cloud, from which we estimate the topological structure using persistent homology (Betti numbers) and the intrinsic dimension (ID). Once the ID of the SWR is determined, dimensionality reduction techniques can be used to embed data into the ID-low dimensional space. **b**, Synthetic SWR were build using three parameters: amplitude, frequency and duration. **c**, Barcodes for the three homology dimensions as calculated for a 2D-torus and synthetic SWR in the 127D-space (2000 events). H0 indicates the number of connected components that persist after increasing R. H1 quantifies the number of loops. H2 identifies the number of cavities. Note different topological features for the torus (1 continuous component, 2 loops and 1 hole), a continuous distribution (80–240 Hz) of synthetic SWR (1 continuous component, no loops, no holes) and a 3-cluster distribution (80–100 Hz; 130–150 Hz; 190–210 Hz) of synthetic SWR (3 continuous components, no loops, no cavities). **d**, Noisy objects (noise equivalent to 0.1 of the object amplitude) were created in 127D to provide ground truth (GT) for ID estimation. Note GT dimension is consistently estimated only with the Angle-based Intrinsic Dimension (ABID) method, while ESS (Expected Simplex Skewness) and PCA (Principal Component Analysis) result in some biases. PCA Local, EES Local and Maximal Likelihood Local (MaxL) all assume that the data is local (that is the curvature and noise within the neighborhood is small) and so they fail to capture GT for challenging objects such as the torus and the Swiss Roll. Instead, ABID is based on angle estimation in the local neighborhood and so it better fits to curved topologies. Bars reflect single values. **e**, Effect of data density (number of SWR events) at the 127D input space in ID estimation for experimental and synthetic SWR, as well as for random LFP segments of similar length. **f**, Effect of the window length in ID estimation of experimental SWR as calculated from EES Local, PCA Local and ABID. Note consistency of ABID estimations. **g**, Effect of the LFP sampling rate in estimation of ID.

To represent SWR variability from different sessions, LFP signals from the SP were filtered (70–400 Hz), *z*-scored and downsampled (2,500 Hz), allowing the projection of individual ripples into a 127-dimensional (127D) space defined around the event peak (±25 ms; one dimension per sample, one point per event; 10,741 events, 58 independent sessions, 27 mice; Fig. 2c and Extended Data Fig. 1a). We deliberately filtered the LFP of all previously validated ripples in a wide frequency range to allow for the evaluation of their feature variability and to ease comparison across species. Ripples would distribute in the high-dimensional space according to their waveform values, reflecting both local and global variations (that is, SWRs of similar frequencies but slightly different amplitudes will fall less apart than SWR of contrasting frequencies). Importantly, here the high-dimensional axes represent temporal LFP samples from one LFP channel, in contrast with the structure of transcriptomic (gene space) or neural manifold (single-cell space) data^24–26^.

First, we sought to examine the topology of the SWR cloud in the 127D space by estimating the presence of discontinuous components, holes and cavities with persistent homology^21^. To this purpose, points (SWRs) within a given radius in the cloud are connected through different simplices consisting of a set of dots, lines, triangles and tetrahedrons (Fig. 2d). Persistent homology looks for the persistence of these connected components (simplicial complexes) as the radius varies, which is quantified with the Betti numbers of the different homology groups (H): H_o_ represents path-connected components, H_1_ refers to loops and H_2_ refers to cavities (Fig. 2d).

We tested the method with a two-dimensional (2D) torus, as well as with synthetic SWRs built from three independent features (frequency, amplitude and duration) in the 127D space (Methods). Synthetic SWRs were built either using a continuous frequency distribution (hence a continuum was expected) or from three separate frequency ranges (hence three separated clusters were expected; Extended Data Fig. 1b). Persistent homology successfully identified their topological features (Extended Data Fig. 1c). That is, a torus in 127D exhibited one cavity and one connected component with two loops, while synthetic ripples showed the expected topologies (either one or three connected components). When applied to the experimental SWRs, the Betti numbers were consistent with a continuous distribution in the 127D space (Fig. 2e), without holes or cavities, suggesting that their classification is not based on discrete categories.

Next, we examined the intrinsic dimension of SWRs in the 127D space, that is, the minimal number of dimensions that preserves data structure. We used a set of methods that relied on measuring the local structure in the neighborhood of each point of the cloud, so that we could infer dimension independently of reconstruction approaches. We tested their performance using ground-truth data (objects in a 127D space) and found the angle-based intrinsic dimension (ABID) method^22^ to provide the most reliable results (Extended Data Fig. 1d). ABID derives the theoretical distribution of angles and uses this to construct an estimator for intrinsic dimensionality. We tested the continuous synthetic events, which exhibited an intrinsic dimension of 3 when estimated with ABID, as expected (Fig. 2f). We found that the 127D cloud of experimental ripples had an intrinsic dimension of 4, similar to the intrinsic dimension of continuous synthetic events with equivalent added noise (Fig. 2f). The intrinsic dimension estimated with ABID was preserved for different window lengths, number of events and sampling rates (Extended Data Fig. 1e–g). Thus, most of the high-dimensional SWR waveform structure could be successfully recovered in a low-dimensional space.

### Low-dimensional embedding of ripple waveforms

**Fig. 3:**
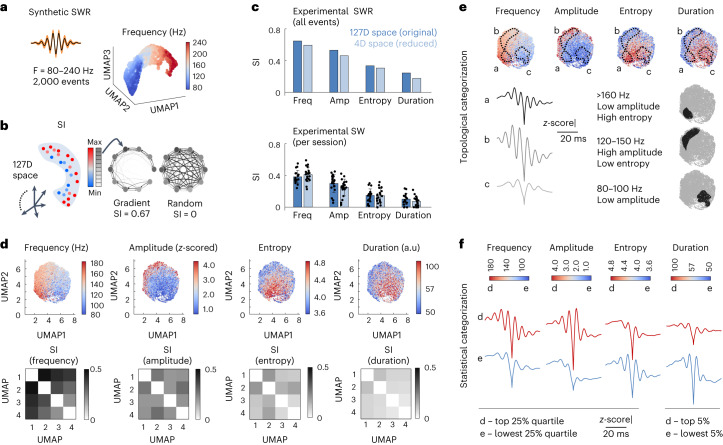
Topological analysis of SWR waveforms. **a**, Synthetic SWRs generated using a continuous distribution of frequency (80–240 Hz), as visualized in a 3D space reduced by UMAP (*n* = 2,000 events). **b**, The SI quantifies the distribution of a given feature (for example, amplitude) over the data cloud (a single value per cloud). Feature values are binned (gray boxes) and the overlap between data points sharing similar bin values is transformed in a weighted directed graph (right). An SI of 1 identifies zero overlap, while 0 identifies a random distribution. **c**, Single values of the SI characterizing the feature distribution of experimental SWRs in the original and the 4D UMAP space. Bars at the top reflect single values of the SI of the data cloud built from all events (10,741 events, 58 independent sessions, 27 mice). Bars at the bottom reflects mean ± s.d. values per session with >200 events (*n* = 19 independent sessions from 15 mice). **d**, UMAP1 and UMAP2 projections of the 4D embedding (top). The SI of each feature per UMAP projection is shown in the matrices below. **e**, Mean ripple waveform of SWRs from different regions of the embedding determined by contour analysis of density distribution in the UMAP plot (Extended Data Fig. 3h). The regions a (1,147 events), b (1,797 events) and c (1,211 events) were defined based on exploratory heuristic criteria to identify SWRs of different conjunctive features. **f**, Statistical categorization of SWRs (top and lowest distribution percentiles). a.u., arbitrary units.

**Extended Data Fig. 2:**
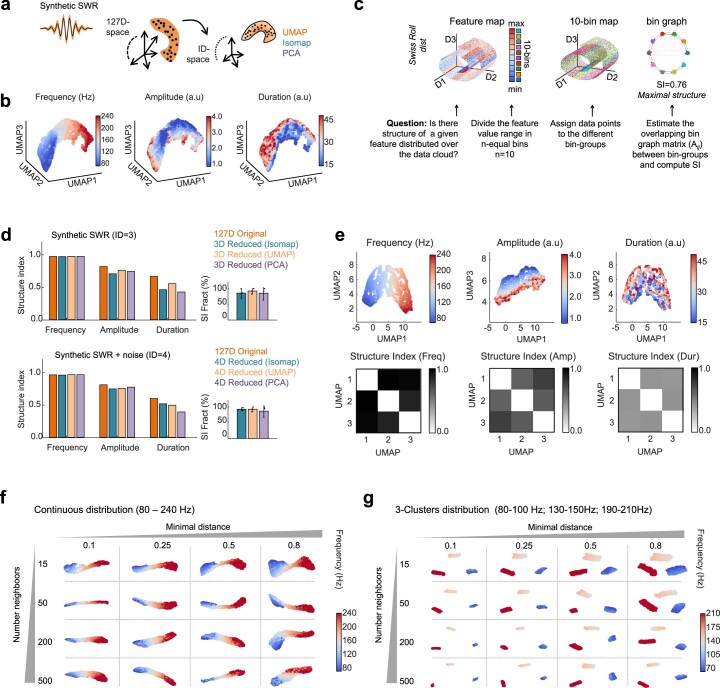
Quantification of SWR feature distribution in the high- and low-dimensional spaces. **a**, Sketch of the dimensionality reduction approach applied to synthetic SWR without noise (ID = 3). **b**, Feature distribution in the 3D embedding of synthetic SWR events without noise as reconstructed with UMAP (2000 events). **c**, Structure Index as a metric to evaluate data feature distribution. The range value of each feature is n-binned (n = 10) and bin-values mapped onto the cloud to identify bin-groups (points sharing similar bin-values). Feature values can be patterned or randomly distributed over the data in any arbitrary dimension. In the example they are distributed with some gradient overlap over a Swiss Roll in a 3D-space. Overlapping between bin-groups was evaluated using graph analysis (see Methods). The Structure Index (SI) takes values between 0 (random feature distribution, fully connected graph) and 1 (maximally separated feature distribution, non-connected graph). Note that this metric can be applied to arbitrary cloud distributions in n-dimensional spaces. **d**, Single values of the SI characterizing the feature distribution of synthetic SWR in the original and 4D UMAP space, built with different methods (Isomap, UMAP and PCA). Bars at left reflect single values of the structure index of the data cloud from all events. Results from synthetic SWR without (top) and with added noise (bottom) are shown separately for the intrinsic dimension of 3 and 4, respectively. Bars at right represent the mean ± SD, with individual data points per feature projected in each space (n = 3 features). Note UMAP maximally retrieves the high-dimensional information from all features at the reduced embedding (SI Fraction recovered from the original space, shown at right). **e**, Two-dimensional projection of the 3D UMAP embedding of synthetic SWR at coordinates with maximal structure per feature (upper plots; that is UMAP1,2 for frequency and duration; UMAP1,3 for amplitude). The Structure Index of each feature per UMAP projection is shown in the matrices below. **f**, Parametric dependence of the shape and orientation of the UMAP reduced embedding, and frequency mapping for a continuous distribution of synthetic SWR (80–240 Hz) without noise. Note that the UMAP embedding is invariant to rotation and translation, with the shape preserved. UMAP reconstruction parameters used by default were 0.1 minimal distance and 15 neighbors. **g**, Same as in f for a clustered distribution of synthetic SWR (80–100 Hz; 130–150 Hz and 190–210 Hz). Note consistent embedding in three separate clusters.

To visualize the SWR cloud, we then applied dimensionality reduction methods, including uniform manifold approximation and projection (UMAP)^27^, Isomap^28^ and principal component analysis (PCA), informed by the intrinsic dimension (Extended Data Fig. 2a). We first tested the continuous synthetic SWRs without noise, which can be reduced to three dimensions (3D), and found striking distribution of events by frequency (Fig. 3a), amplitude and duration (Extended Data Fig. 2b; for UMAP as an example). This suggests that events are mapped into the high-dimensional waveform space according to a nontrivial distribution that maximizes the independent structure of their characteristic features. To quantify this property, we used a graph-based index (structure index, SI) that evaluates the overlap between feature values projected over the data cloud (Fig. 3b and Extended Data Fig. 2c)^23^. For example, a perfect gradient distribution of a given feature will give SI values close to 1, while a random distribution will give values close to 0.

Using this index, we examined how much structure can be obtained per feature in the original and the reduced space. For synthetic SWRs, UMAP provided reconstructions with feature distributions more similar to those in the original space than Isomap and PCA (Extended Data Fig. 2d). 2D projections of the 3D cloud confirmed variations of feature distribution along UMAP axes (Extended Data Fig. 2e). While the UMAP embedding can be subject to translation and rotation, the overall shape and feature distribution was consistent across reconstruction parameters for both continuous and discontinuous synthetic SWRs (Extended Data Fig. 2f,g).

**Extended Data Fig. 3:**
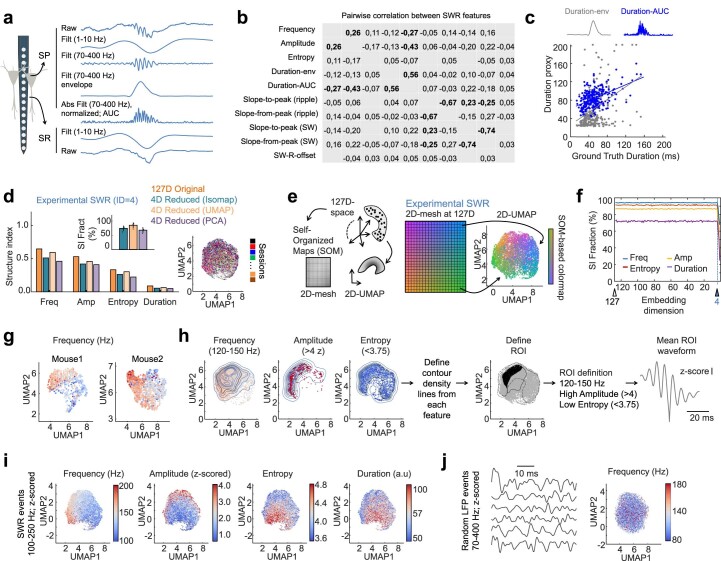
Experimental SWR feature analysis. **a**, Processing of experimental LFP signals for estimating different SWR features. Raw signals at SP (defined from the maximal ripple power) and SR (sharp-waves) were filtered in different bands to define amplitude (70–400 Hz envelope), slopes (1–10 Hz) and duration-env, from the 70–400 Hz envelope at the mean amplitude; duration-AUC from the area under the curve of the amplitude normalized signal). Spectral features (frequency and entropy) were obtained from the individual SP spectra. The frequency was estimated from the bump spectral peak of each SWR event. The entropy was defined after normalizing the entire spectra (area = 1) binned at 10 Hz. Slopes from the sharp-wave were calculated from the SR filtered signal. See Methods for details. **b**, Pearson R-values between features exhibiting significant correlation (p < 0.001; two-sided). Significant R-values above/below ±0.25 are shown in bold. **c**, Correlation between the two measurements of ripple duration (Duration-env and Duration-AUC) as compared against ground truth (SWR starts and ends were manually evaluated by an expert; see Methods). We found that defining the ripple duration from the AUC provided best correlation with the ground truth. Note the two measures correlated significantly between each other (R = 0.56; p < 0.00001 as shown in b). **d**, Single values of the SI characterizing the feature distribution of experimental SWR in the original and 4D space built with different methods (Isomap, UMAP and PCA). Bars reflect single values of the structure index of the data cloud from all events. At inset, bars reflect mean ± SD of the fraction of structure from the original space that is lost in the 4D space built with different methods using data from all features (n = 4 features per method). Note UMAP maximally retrieves the high-dimensional information at the reduced embedding. The UMAP1,2 projection per session is shown at right. **e**, Comparison with Self-Organizing Maps (SOM). SOM acts to fit a 2D-mesh to the data cloud in the original 127D-space. A 2D-UMAP was built similarly and the distribution of SWR was compared by using SOM-based colormaps. Note roughly similar organization of events in the 2D-mesh and the 2D-UMAP. **f**, Structure index fraction recovered from the original space while embedding experimental SWR with UMAP at progressively lower dimensions. Note that the feature structure is maximally retrieved with UMAP up to the estimated intrinsic dimension of 4, suggesting suboptimal representations with 2D-UMAP. **g**, UMAP embedding from SWR from two different mice (mouse1: 808 events; mouse2: 1248 events). Note similar distribution invariant to rotation and translation in the UMAP coordinates. **h**, Definition of Region of Interests (ROIs) in the UMAP embedding using heuristic criteria. Contour lines are defined based on the density of points (SWR) sharing particular feature values. In the example, SWR of 120–150 Hz are identified and the contour density lines are defined from the probability density distribution. Same for SWR of > 4 z-scored amplitude and >3.75 entropy values. The ROI is defined from the overlapping region. **i**, UMAP projections of SWR detected in the 100–250 Hz band. **j**, UMAP projection of random LFP events. Note lack of structure for frequency.

We next examined the organization of experimental SWR events using different features estimated from LFP traces (Extended Data Fig. 3a,b; that is, ripple frequency, spectral entropy and duration; Table 1). Maximal structure emerged consistently for frequency in both the original and the four-dimensional (4D) UMAP space, followed by amplitude, entropy and a proxy for duration (Fig. 3c), the latter calculated from an extended window around the event and expressed in arbitrary units (Extended Data Fig. 3c). We also noted structured distribution of some nonspectral features such as the slope of the event defined from the SWR peak (Table 1). In all cases, UMAP outperformed other methods in recovering information from the original space, with SWRs from different experimental sessions contributing similarly (Fig. 3c, bottom; Extended Data Fig. 3d, right). When compared with a previously used 2D reduction method^7,18^, UMAP yielded comparable results (Extended Data Fig. 3e), but as expected from the intrinsic dimension, optimal recovery of information from the high-dimensional space required at least four dimensions (Extended Data Fig. 3f). SWRs recorded from individual mice exhibited similar trends (Extended Data Fig. 3g).

**Table 1:** SI of SWR features over the waveform space

All events						
	Original space 50 ms	Reduced space 50 ms	Original space 100 ms	Reduced space 100 ms	Original space 200 ms	Reduced space 200 ms
**Frequency**	**0.64**	**0.59**	**0.62**	**0.56**	**0.59**	**0.56**
**Amplitude**	**0.53**	**0.46**	**0.47**	**0.43**	**0.43**	**0.43**
**Entropy**	**0.33**	**0.30**	**0.32**	**0.30**	**0.30**	**0.27**
Duration-env	0.09	0.06	0.11	0.07	0.10	0.05
**Duration-AUC**	**0.24**	**0.17**	**0.25**	**0.17**	**0.24**	**0.20**
Slope-to-peak (ripple)	0.16	0.13	0.04	0.03	0.02	0.02
Slope-from-peak (ripple)	0.12	0.10	0.07	0.05	0.04	0.03
Slope-to-peak (SW)	0.10	0.08	0.09	0.08	0.09	0.08
**Slope-from-peak (SW)**	**0.13**	**0.11**	**0.13**	**0.11**	**0.10**	**0.10**
SWR offset	0.03	0.03	0.04	0.04	0.05	0.03
Per session						
**Frequency**	0.3802****	0.4091****	0.3485****	0.3668****	0.3381****	0.3617****
**Amplitude**	0.2968****	0.2466****	0.2694****	0.2481****	0.2665****	0.2805****
**Entropy**	0.1463****	0.1426****	0.1301****	0.1467****	0.1148****	0.1251****
Duration-env	0.0260****	0.0198****	0.0192****	0.0210	0.0138****	0.0146
Duration-AUC	0.0997****	0.0719****	0.0919****	0.0751****	0.0904****	0.0874****
Slope-to-peak (ripple)	0.0916****	0.0883****	0.0141****	0.0160	0.0016	0.0052
Slope-from-peak (ripple)	0.0671****	0.0681****	0.0206****	0.0272****	0.0235****	0.0288****
Slope-to-peak (SW)	0.0471****	0.0437****	0.0547****	0.0540****	0.0494****	0.0552****
Slope-from-peak (SW)	0.0688****	0.0724****	0.0692****	0.0785****	0.0689****	0.0848****
SWR offset	0.0374****	0.0347****	0.0477****	0.0482****	0.0417****	0.0467****

Data for all events represent single values of the SI calculated over the entire data cloud (10,741 events, SWRs, all sessions) in the original and the 4D UMAP space built with different windows lengths. Data per session represent the mean value of the SI over the ripple cloud calculated per sessions with >200 events (*n* = 19 independent sessions). Bold values indicate consistent structure (SI > 0.1) in the original and the reduced space across windows.

Values were significantly different than shuffled distribution at ****P < 0.0001 (one-sample one-sided *t*-test). AUC, area under the curve; Env, envelope.

Visualization of feature variability across UMAP projections confirmed the continuous organization of experimental SWRs, consistent with results from persistent homology (Fig. 3d). High-amplitude and low-amplitude events distributed all along the frequency gradient, with different trends for entropy and duration. These nontrivial interdependencies between SWR features cannot be captured by linear correlation analysis (Extended Data Fig. 3b). Instead, analysis along the embedding allowed for a richer, heuristic, categorization of SWRs (Fig. 3e) than that resulting from standard percentile distribution of individual features (Fig. 3f). For instance, using contour analysis of event density in the embedding (Extended Data Fig. 3h), the region of low-amplitude/high-entropy SWRs of >160 Hz (a) can be separated from that of high-amplitude/low-entropy SWRs in the 120–150 Hz range (b) or from low-amplitude SWRs of 80–100 Hz (c; Fig. 3e). Strikingly, all of them emerged from a continuum. We will use these regions as examples of how the method can be applied to better understand SWR mechanisms.

**Extended Data Fig. 4:**
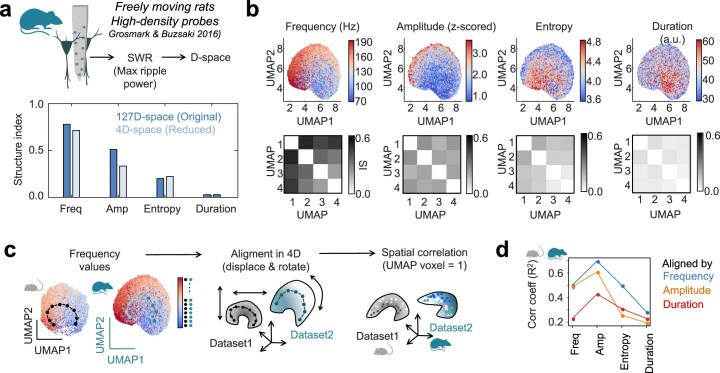
Comparisons between datasets across species. **a**, Analysis of SWR events recorded in freely moving rats with high-density probes in an external dataset. The channel with the maximal ripple power was selected for topological data analysis. Note similar structure distribution per feature than in head-fixed mice, as quantified by the Structure Index (bottom) in the original and the reduced space (intrinsic dimension 4, as estimated by ABID). Bar reflect single values of the SI of the data cloud from all events. **b**, UMAP1,2 projections built from the SP channel with the maximal ripple power. The Structure Index matrices for each feature are shown at bottom. **c**, The 4D frequency representations of the two datasets (mouse head-fixed and rat freely moving) are aligned using feature values at UMAP coordinates. To this purpose the center of mass of points (SWR) with similar feature values (20 bins) were estimated and used for alignment in the 4D reduced space. Once aligned by a given feature, the spatial correlation between features in the two datasets were estimated (UMAP voxels of 1 corresponding to 200 SWRs). **d**, Spatial correlation coefficient (all significant at p > 0.001, two-sided) between features per alignment supporting quantitatively similar distributions in mice and rats. The best alignment strategy of the two embeddings is by frequency. Aligning by entropy yielded no significant correlation and it is not shown.

Similar figures were obtained for SWRs in the standard 100–250 Hz frequency range used in rodent literature^2^ (Extended Data Fig. 3i), while random LFP events containing no ripples failed to show any structure (Extended Data Fig. 3j). SWRs recorded in freely moving rats with high-density probes^29^ (external dataset) showed a similar distribution than for SWRs recorded in head-fixed conditions (our data), as quantified by embedding alignment of the two datasets (Extended Data Fig. 4).

The method and analytical steps leading to these results are illustrated in the following interactive code notebook, which can be executed online: https://colab.research.google.com/drive/1AHG4UQ15NobY2tI7Kc3hQFEkocdRzIsa?usp=share_link#scrollTo=GI8nBd8hOuSv (Code availability).

### Input mechanisms underlying a diversity of ripple waveforms

The results above suggest that variations of SWR features are coherently represented in the high-dimensional and the low-dimensional waveform space. Are there circuit mechanisms underlying the distribution of SWRs along a continuum?

**Fig. 4:**
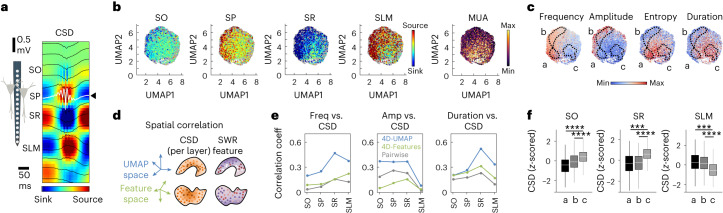
Input mechanisms underlying the distribution of SWR features. **a**, SWR-associated CSD signals estimated from linear silicon probes. Arrowhead marks the SP channel used for topological analysis. Color scale indicates the intensity of sinks (blue) and sources (red). **b**, Distribution of CSD sink and source intensities per SWR confirms that the embedding built from SP signals contains layer-resolved information of the underlying input generators. MUA values were also distributed over the UMAP cloud (color scale from 0 to 0.07 spectral power 300–400 Hz). Data from 2,613 events, 17 independent sessions and 9 mice met CSD criteria. **c**, SWR features and event categorization are shown in Fig. 3e (a, b and c) for comparison with the CSD distribution. The color scale indicates minimal and maximal feature values as shown in Fig. 3d. **d**, Spatial correlation between SWR features and CSD values was calculated in a 4D space using voxels. The 4D space was defined by either the UMAP coordinates (blue axes) or by SWR features (green axes). In both cases, the SWRs will form a point cloud, but they will differ in their shapes. **e**, Significant correlation coefficients between SWR features and CSD values as estimated pairwise comparisons (gray) and from UMAP (blue) and feature (green) spaces. **f**, Box plots show median CSD values at the SO, the SR and the SLM for the different categories of events as horizontal lines, with the first and third quartiles denoting box limits (a, 1,147 events; b, 1,797 events; and c, 1,211 events). Whiskers indicate the data point farthest from the quartile values that is within 1.5 times the interquartile range. One-way ANOVA per layer: SO *F*(2) = 67.3; SR *F*(2) = 102.3; SLM *F*(2) = 61.4, all at *P* < 0.0001. Post hoc Tukey–Kramer two-tailed tests ***P < 0.001; ****P < 0.0001.

To gain mechanistic insights, we next estimated the current source density (CSD) signals of individual SWRs using all channels from the recording probe (Fig. 4a; 2,613 events, 17 independent sessions from 9 mice meeting CSD criteria), as well as the associated multiunit activity (MUA) firing from the cell body layer. A CSD sink (blue) corresponds to active depolarizing currents driven by glutamatergic input pathways at the specific hippocampal strata, while a CSD source (red) could be interpreted either as the passive return current or as an active hyperpolarization driven by GABAergic inhibitory inputs.

**Extended Data Fig. 5:**
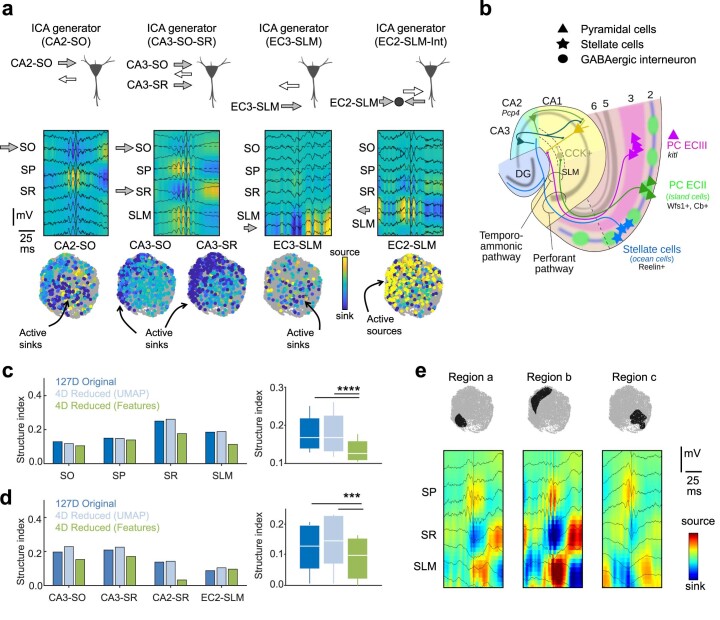
Input pathway generators. **a**, Independent component analysis (ICA) of SWR-associated current generators were estimated by spatial discrimination of LFP signals in a subset of experiments meeting methodological criteria for CSD and ICA reconstruction. Each generator was identified according to existing knowledge on pathway-specific sinks and sources (see b). The CA2 generator was heuristically identified from ICA components exhibiting a SWR-associated sink at SO and a source at SP/SR border (406 SWR from 5 sessions). The CA3 SWR generator was associated with sinks at SO and SR, which are shown separately (625 SWR from 7 sessions). Similarly, we identified two entorhinal cortex (EC) generators, associated to the layer 3 direct glutamatergic input pathway (EC3-SLM; 173 SWR from 4 sessions) and the indirect inhibitory EC2 inputs (EC2-SLM; 585 SWR from 7 sessions), via CCK interneurons. The location of the active sinks and sources from each generator is indicated in the embedding. **b**, Summary of knowledge base used to inform identification of ICA generators. Note the two different EC input pathways associated with active sinks (EC3) and sources (EC2 via feedforward inhibition), which are located at two different depths into SLM. **c**, Distribution of CSD values per layer in the 127D original space, the 4D-UMAP space and the 4D-Feature space was evaluated using the structure index. Bars at left reflect single values per layer. Note similar distribution in the original and the UMAP space, and lower structure index values for all layers in the 4D-feature space. Box plots at right show the median structure index (horizontal bars) per space for all layers as data points (n = 4), with the first and third quartiles as box limits. Whiskers indicate the data point further from quartile values that is within 1.5 times the interquartile range. Significant differences between the feature space and each of the topological spaces are indicated (****p < 0.0001; two-sided Student t-test). **d**, Structure index of the distribution of CSD values per ICA generator in the 127D original space, the 4D-UMAP space and the 4D-Feature space. Bars at left reflect single values per generator. The EC3-SLM generator is not shown due to poor sampling. Box plots at right show the median structure index per space (horizontal lines) for all generators as data points (n = 4), with the first and third quartiles as box limits. Whiskers indicate the data point further from quartile values that is within 1.5 times the interquartile range. Significant differences between the feature space and each of the topological spaces are indicated are indicated (***p < 0.001; two-sided Student t-test). **e**, CSD maps of SWR events in region a, b and c, defined according to exploratory heuristic criteria.

We projected MUA and CSD values over the embedding that resulted from SP ripples (Fig. 4b). Strikingly, CSD values from different layers segregated along the embedding, suggesting that ripple waveforms are carrying latent laminar information about the assortment of synaptic inputs. By confronting the distribution of CSD and MUA values with that of SWR features, and the previously defined regions (a, b and c), we noted some remarkable trends (Fig. 4b versus 4c). For example, MUA values distributed similarly to the spectral entropy, consistent with population firing leaking into the ripple band. Interestingly, the distribution of the SR sinks (for example, CA3 inputs) seemed to follow that of ripple frequency and amplitude, whereas CSD values at the stratum oriens (SO; for example, CA2 and CA3 inputs) and the stratum lacunosum moleculare (SLM; entorhinal inputs) seemed to be associated with the distribution of SWR amplitude and duration, respectively. Similar trends were appreciated for input-specific generators (CA3, CA2 and entorhinal inputs from layers 3 and 2), estimated with independent component analysis (ICA; Extended Data Fig. 5a,b).

We confirmed some of these intuitions by calculating the spatial correlation between CSD values and SWR features for the same set of events, using voxels in the 4D UMAP space (Fig. 4d and Methods). Spatial correlation extracted more structure than direct pairwise comparisons between SWR feature values (Fig. 4e; blue and gray traces, respectively). To evaluate whether the low-dimensional waveform space provided more information as compared with simpler approaches, we also looked at the spatial correlation between CSD values and SWR features projected in a feature space (that is, the 4D space made of frequency, amplitude, entropy and duration; Fig. 4d). We found less spatial correlation in a 4D space built from the predefined features versus that resulting from the embedded waveform space (4D UMAP), and even for pairwise comparisons (Fig. 4e). This is because the spatial correlation in the reduced waveform space takes into account the topological organization of events in the voxel neighborhood, in contrast to the feature space. Consistently, the SI of CSD values at the feature space was lower than in the original and the reduced waveform space (Extended Data Fig. 5c; see the same for ICA components in Extended Data Fig. 5d).

According to spatial correlation analysis, the organization of ripples in the waveform space is mostly determined by the assortment of inputs. Inputs arriving at the SR (that is, CA3) mostly explain the distribution of SWRs in the waveform space according to their frequency and duration, while their distribution by amplitude is determined by SO and SR inputs (that is, CA2 and CA3). This is consistent with a CA2 and CA3 origin of different types of SWR events^30,31^. Instead, entorhinal cortical inputs at the SLM may influence the distribution of SWRs according to their frequency and duration, but not their amplitude, consistent with previous data^30,32^. No significant spatial correlation was found between the distribution of CSD and entropy values in the waveform space (all layers at *P* > 0.05). In contrast, events with higher MUA values distributed closer to those with higher spectral entropy (correlation coefficient *R*^2^ = 0.61) and lower amplitude (*R*^2^ = 0.19; both at *P* < 0.0001). Importantly, we confirmed different contributions of associated sinks and sources in shaping the previously topologically categorized SWR events a, b and c (Fig. 4f and Extended Data Fig. 5e), permitting physiological interpretation. For instance, while high-amplitude/low-entropy SWRs of 120–150 Hz (region b) were associated with the typical large SR sink and SLM sources, low-amplitude SWRs of 80–100 Hz (region c) instead exhibited sinks at the SLM in association with sources at the SR (Extended Data Fig. 5e).

### Optogenetic validation of the low-dimensional embedding

**Fig. 5:**
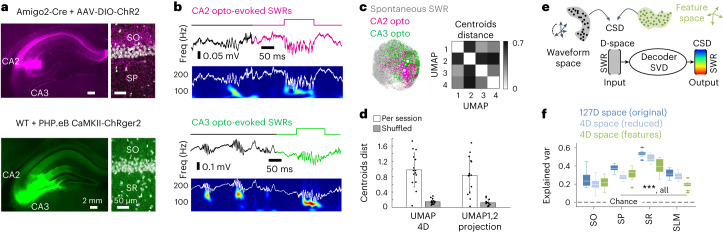
Optogenetic validation. **a**, Representative images of transgenic and viral strategies used for specific expression of channelrhodopsin in CA2 and CA3 terminals. All animals were histologically validated. **b**, Examples of SWRs evoked in CA1 by optogenetic activation of CA2 and CA3 terminals. **c**, Mapping of evoked SWRs onto the UMAP1/2 projection. Centroids from independent sessions (small dots; CA2: 5 sessions, 4 mice; CA3: 3 sessions, 2 mice) and for all sessions together (large dots; CA2: 1,220 events; CA3: 1,715 events) are shown. The distance between centroids on all UMAP projections is shown to the right. Note the diagonal is set to zero. **d**, Centroids distance between CA3-evoked and CA2-evoked SWRs per independent session tested significantly against shuffled distributions. Bars represent the mean ± s.d. of CA3–CA2 centroid distances from all possible combinations between sessions (*n* = 15 combinations from 5 × 3 sessions from 6 mice). **e**, Decoding strategy using SWRs coordinates in a D-space to infer the associated CSD signals. Results from the original (127D) and reduced (4D UMAP) waveform space were compared with those resulting from a 4D feature space. A SVD was trained and tested using a tenfold strategy. **f**, The explained variance of CSD values per layer as predicted by the SVD in the test set. Box plots show the median explained variance (horizontal line) at all layers for each space resulting from the tenfold prediction (*n* = 10 tests), with the first and third quartiles as box limits. Whiskers indicate the data point farthest from quartile values that is within 1.5 times the interquartile range. Two-way ANOVA effects for layer (*F*(3,2) = 131.4), reconstruction space (*F*(3,2) = 30.2) and interaction (*F*(3,2) = 4.9), all at *P* < 0.0001. Post hoc Tukey–Kramer two-tailed tests all at ***P < 0.001. The upper limit of chance level as estimated from shuffled data is indicated (discontinuous line).

**Extended Data Fig. 6:**
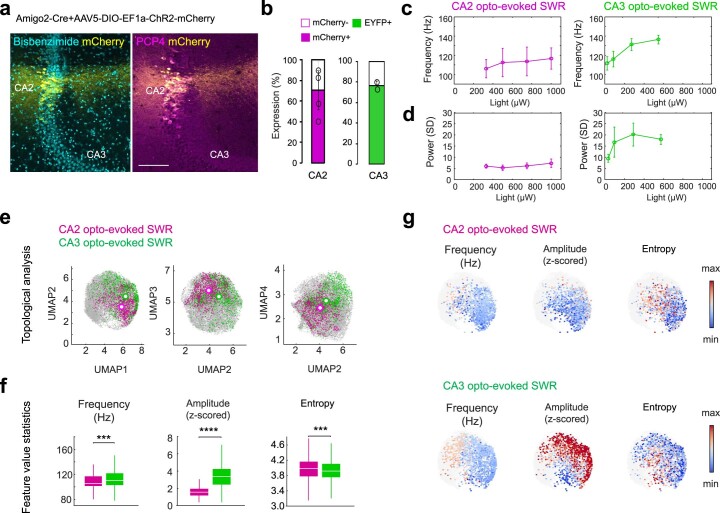
Input dissection with optogenetics. **a**, Immunostaining validation of channelrhodopsin (ChR2) expression specificity (mCherry) in the Amigo2-cre line used to target CA2 pyramidal cells (PCP4+). Scale bar correspond to 130 µm. **b**, Quantification of ChR2 expressing cells (mCherry+) among PCP4+ CA2 pyramidal cells (mean ± SD data from 4 sections from 4 mice). At right, quantification of CA3 specificity of channelrhodopsin expression obtained with the AAV PHP.eB CaMKII-ChRger2-EYFP strategy (expressed as percentage of EYFP+ pyramidal cell layer along the CA3 region defined from CA3c to CA3a at the border with CA2) (mean ± SD data from 2 histological sections from 2 mice). **c**, Dependence of the frequency of SWR evoked optogenetically from CA2 and CA3 terminals. Note relatively constant ripple frequency, independent of light stimulation intensity for CA2 but not for CA3 evoked SWR, consistent with results from spatial correlation. Plots show the mean ± SD frequency of evoked SWR per light intensity for CA2 (n = 3 sessions, 3 mice) and CA3 terminals (n = 3 sessions, 2 mice), separately. **d**, Same as in d for the ripple power. **e**, Topological analysis of optogenetically evoked SWR. The centroid of each experimental group is shown in different UMAP projections. **f**, Feature value statistics of optogenetically-evoked SWR. Box plots show the median feature value for CA2 (n = 1220 events from 5 sessions, 3 mice) and CA3 evoked SWR (n = 1715 events from 3 sessions, 2 mice) as horizontal bars, with the first and third quartiles as box limits. Whiskers indicate the data point further from quartile values that is within 1.5 times the interquartile range. Given the evoked nature of the events, duration is not reported. Two-sided Student t-test, ***, p < 0.001; ****, p < 0.0001. **g**, Distribution of feature values of optogenetically-evoked SWR over the UMAP1,2 projection. Note consistent feature distribution over the embedding as compared with spontaneous SWR and differences between CA2- and CA3-triggered events.

To better explore these ideas and to improve interpretation, we sought to examine the topological distribution of SWRs generated by CA3 and CA2 (ref. ^31^). Thus, we expressed channelrhodopsin in upstream CA3 and CA2 pyramidal cells using transgenic and viral strategies (Fig. 5a and Extended Data Fig. 6a,b). We mimicked the CA2-specific and CA3-specific prolonged synaptic release that accompanies SWRs by using green-light pulses of 100-ms duration, which mildly activate channelrhodopsin currents (Methods). Consistently, optogenetic activation of these terminals resulted in evoked SWRs of different features in CA1 (Fig. 5b).

In agreement with correlation analysis, we noted that the frequency and amplitude of CA2-evoked events could not be modulated by increasing the light power, in contrast to CA3-evoked SWRs (Extended Data Fig. 6c,d). To compare with spontaneous events, we isolated evoked SWRs in windows around their power spectral peaks (±25 ms), as before. Strikingly, optogenetically evoked SWRs fitted differently across the UMAP embedding (Fig. 5c and Extended Data Fig. 6e). CA3-evoked events spread toward the region of high-amplitude/high-frequency (for example, region b), while CA2-evoked SWR events remained more confined toward the low-frequency/low-amplitude region (for example, region c). These results did not simply reflect differences on the mean frequency of evoked SWRs (Extended Data Fig. 6f), instead all feature values were consistently distributed in the UMAP embedding (Extended Data Fig. 6g). The different distribution of CA2-evoked and CA3-evoked SWRs was confirmed by computing the distance between centroids across all UMAP projections (Fig. 5c; 1,220 events from CA2; 1,715 events from CA3), with centroid distance per projection/session tested significantly against the shuffled distribution (Fig. 5d). Importantly, the distribution of CA3-evoked and CA2-evoked SWRs over the UMAP embedding resembled the region where ICA localized their associated active synaptic sinks (Extended Data Fig. 5a).

**Extended Data Fig. 7:**
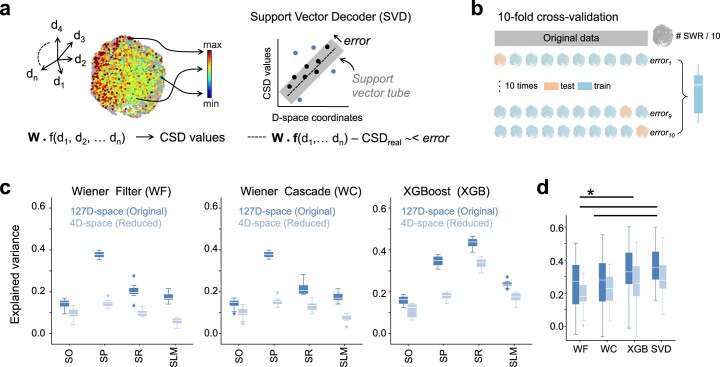
Topological decoding. **a**, Schematic representation of the decoding strategy to predict CSD values from the SWR space. In any arbitrary space, SWR occupies different positions along the cloud. Each position is associated with specific CSD values. Any decoder seeks to map the real values of CSD into the representational space, so that they can be unambiguously predicted. A Support Vector Decoder (SVD; right) is a regression algorithm that looks to minimize the error tube, instead of seeking the best curve for a decision boundary. SVD find the closest match between data points and the mapping function. **b**, Schematic representation of the 10-fold cross-validation strategy. The SWR data cloud is divided in 10 samples. Nine of these samples are used for training and the remaining for testing; an error is computed. The procedure is repeated 10 times, providing mean results. **c**, CSD explained variance as predicted from the Wiener Filter, Wiener Cascade and XGBoost models of SWR mapped into the D-dimensional space (127D original and 4D reduced). Results from the Support Vector Decoder (SVD) are shown in the main figure. Box plots show the median explained variance (horizontal line) at all layers per space for the 10-fold prediction (n = 10 tests), with the first and third quartiles as box limits. Whiskers indicate the data point further from quartile values that is within 1.5 times the interquartile range. **d**, Comparison between decoders in the original and the reduced space. Data per layer was aggregated to estimate the mean explained variance per decoder and tested with two-way ANOVA. Box plots show the median explained variance (horizontal line) per decoder resulting from aggregating data from all layers (n = 4), with the first and third quartiles as box limits. Whiskers indicate the data point further from quartile values that is within 1.5 times the interquartile range. Effects for decoders (F(3,1) = 13.9, p < 0.00001) and input space (F(3,1) = 20.5, p < 0.00001). Posthoc Tukey-Kramer two-tailed tests *, p < 0.05. The SVD was chosen for all simulations, given maximal mean values.

Overall, these results suggest that layer-resolved information of individual ripples is represented in both the high-dimensional and the low-dimensional spaces built from SP signals. We therefore trained a support vector decoder (SVD) to infer CSD values using only the position of spontaneous SWRs in the input space (Fig. 5e; tenfold design for training and test; see Extended Data Fig. 7 for details and results from other decoders). We found that this strategy successfully explained a large part of the CSD variance (Fig. 5f). While results were in general better using data from the high-dimensional space, trends were best preserved using coordinates of the 4D UMAP space as compared with the feature space. Actually, an SV classifier operating over the 4D UMAP space successfully identified evoked SWRs from CA3 and CA2 at 0.65 accuracy, well above chance level (*P* < 0.0001) and independently on differences of frequency and amplitude (0.67 and 0.62 accuracy for SWR events equalized by frequency and amplitude, respectively).

Thus, our topological and low-dimensional analysis of ripple waveforms can provide mechanistic interpretation of SWR feature variations depending on CA1 microcircuit activation by different input pathways. Importantly, this strategy may allow for inference of the underlying mechanisms from single-channel recordings even in the absence of precise laminar information.

### Effects of brain states and cognitive demands

**Fig. 6:**
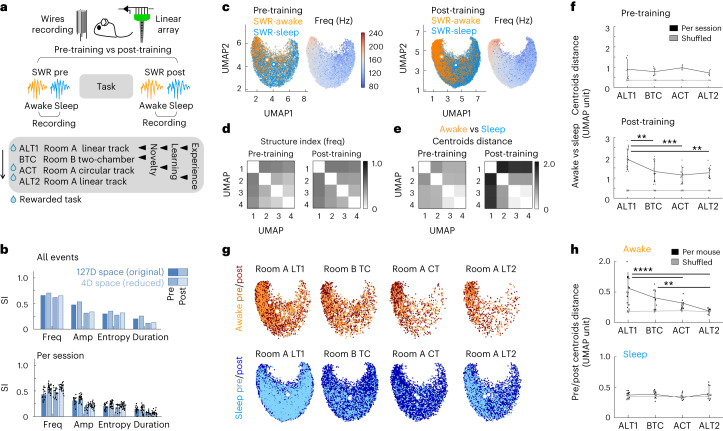
Topological analysis of state and cognitive influences on SWRs. **a**, Multiple tasks evaluated the effect of novelty, learning and experience. Mice implanted with wires experienced the tasks in the same order across days. Mice recorded with silicon probes performed only the first task and were used for topological analysis and decoding. SWRs were recorded over 2 h before and after each task. **b**, SI per feature before and after tasks, as estimated from the original and the reduced space, for all events together at top (single values) and for each pre-/post-training session with >200 events at bottom (mean ± s.d; pre: *n* = 12 independent sessions, 7 mice; post: *n* = 22 independent sessions, 8 mice). **c**, UMAP1/2 projection from the 4D embedding built from SWRs recorded pre-training (left) and post-training (right) (all tasks). Note centroid separation of awake and sleep SWRs. Frequency distribution is shown to the right. **d**, SI for the frequency distribution per UMAP projection of SWRs before and after training. **e**, Distance between awake and sleep SWR centroids before and after training in all UMAP projections. Note maximal structure and centroid separation along UMAP1 projections. **f**, Distance between awake and sleep SWR centroids per session with more than 50 events in both conditions before (top) and after (bottom) the different tasks. Plots reflect the mean ± s.d. centroid distance for all possible combinations of sessions and the three UMAP1 projections (pre: *n* = 9 combinations for ALT1, *n* = 6 for BTC, *n* = 3 for ACT and *n* = 3 for ALT2; post: *n* = 21 for ALT1, *n* = 18 for BTC, *n* = 15 for ACT and *n* = 15 for ALT2). Data were bootstrapped (black), and tested against the shuffled distribution (100 shuffles, gray). Effects for task (post-training): one-way ANOVA *F*(3) = 22.8, *P* < 0.0001. Post hoc Tukey–Kramer two-tailed tests, **P < 0.01; ***P < 0.001. **g**, Topological distribution of pre-/post-training SWR across tasks. Note different trends of pre/post centroid separation for awake and sleep SWR. **h**, Distance between pre/post SWR centroids recorded in awake (top) and sleep (bottom) across tasks, calculated as in **f**. Plots reflect the mean ± s.d. centroid distance for all possible combinations of sessions and the three UMAP1 projections (awake: *n* = 21 combinations for ALT1, *n* = 9 for BTC, *n* = 12 for ACT and *n* = 9 for ALT2; sleep: *n* = 12 for ALT1, *n* = 9 for BTC, *n* = 6 for ACT and *n* = 9 for ALT2). Data were bootstrapped (black), and tested against the shuffled distribution (100 shuffles, gray). Effect for task (awake): one-way ANOVA *F*(3) = 25.8, *P* < 0.0001. Post hoc Tukey–Kramer two-tailed tests **P < 0.01; ****P < 0.0001.

**Extended Data Fig. 8:**
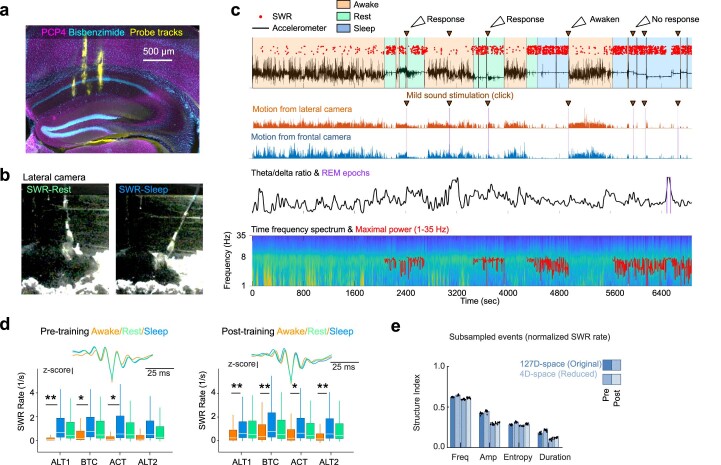
Analysis of SWR recorded from freely moving mice. **a**, Histological validation of silicon probe tracks implanted chronically in freely moving mice. PCP4 immunostaining was used to delineate the border with CA2. Probe and electrode tracks were validated in all mice. **b**, Images from the lateral cameras used to validate the identification of Rest and Sleep states in the home cage. **c**, State scoring approach. Information from the lateral and ceiling cameras were used to validate movement indices calculated from the head-stage accelerometer. The theta/delta signal was estimated from the time frequency spectrum. First, periods of immobility were separated from periods of running (Awake). Immobility periods were classified as Rest (no movement awake) and Sleep based on spectral criteria. The maximal power in the 1–35 Hz band was used to identify REM sleep. Sensory threshold along sleep were tested with mild sound stimulation (clicks; arrowheads), which provided ground truth to separate periods of Rest and Sleep during immobility. SWR detected in the different periods were classified accordingly. **d**, Quantification of the rate of SWR recorded in Awake, Rest and Sleep conditions, both Pre- (left) and Post-training (right). The mean SWR waveform from each state is also shown at the top. Only mice recorded with wires and tested in all tasks were included in the analysis (4 sessions, 6 mice). ALT1: Room A Linear Track 1, BTC: Room B Two-chamber; ACT: Room A Circular Track; ALT2: Room A Linear rack 2. Box plots at left show the median SWR rate at Pre-training per state and task as horizontal bars (n = 510/1824/496 events from Awake/Rest/Sleep in ALT1; n = 821/1972/1141 events from Awake/Rest/Sleep in BTC; n = 417/555/1133 events from Awake/Rest/Sleep in ACT; and n = 425/1774/361 events from Awake/Rest/Sleep in ALT2). Box plots at right show the same for Post-training (n = 1834/2700/2482 events from Awake/Rest/Sleep in ALT1; n = 1692/8706/1154 events from Awake/Rest/Sleep in BTC; n = 1007/5227/1299 events from Awake/Rest/Sleep in ACT; and n = 834/4599/1070 events from Awake/Rest/Sleep in ALT2).The first and third quartiles as box limits. Whiskers indicate the data point further from quartile values that is within 1.5 times the interquartile range. Significant effect of state in a two-way ANOVA for Pre (F(3,2) = 22.9, p < 0.0001) and Post events (F(3,2) = 60.3, p < 0.0001). Asterisks indicate significant differences of post-hoc Tukey-Kramer two-tailed tests: *, p < 0.05; **, p < 0.01. **e**, Structure index per feature after normalizing by same number of SWR events (15144) before and after training (bootstrapped, 10 samples). Bars show the mean ± SD structure index per feature for each sample (n = 10 bootstrapped samples).

Inspired by these ideas, we sought to evaluate how cognitive demands (novelty, learning) and brain state (wakefulness, sleep) influence the expression of SWRs. A long-standing question in the field is what determines differences between SWRs in awake and sleep conditions^5^. With the aim to compare awake versus sleep SWR preceding and following a series of cognitive tasks, we recorded from mice exposed to novel or familiar contexts (rooms A and B, 6 mice implanted with wires), while they were trained for the first time to alternate for water reward in either linear tracks (LTs) or semicircular tracks (CTs), or allowed to explore a two-chamber (TC) field (Fig. 6a). The order of the tasks was the same for all animals. SWRs were recorded in the home cage before and after each task. To provide additional data for training the topological decoder, three additional mice were recorded with linear arrays during the first task only (Extended Data Fig. 8a). SWRs (59,907 events) were classified as belonging to rest (immobility; 11,593 events), awake (exploratory pauses; 9,164 events) and sleep states (non-rapid eye movement (REM) sleep; 39,149 events; data 36 sessions from 9 mice; Extended Data Fig. 8b,c).

**Extended Data Fig. 9:**
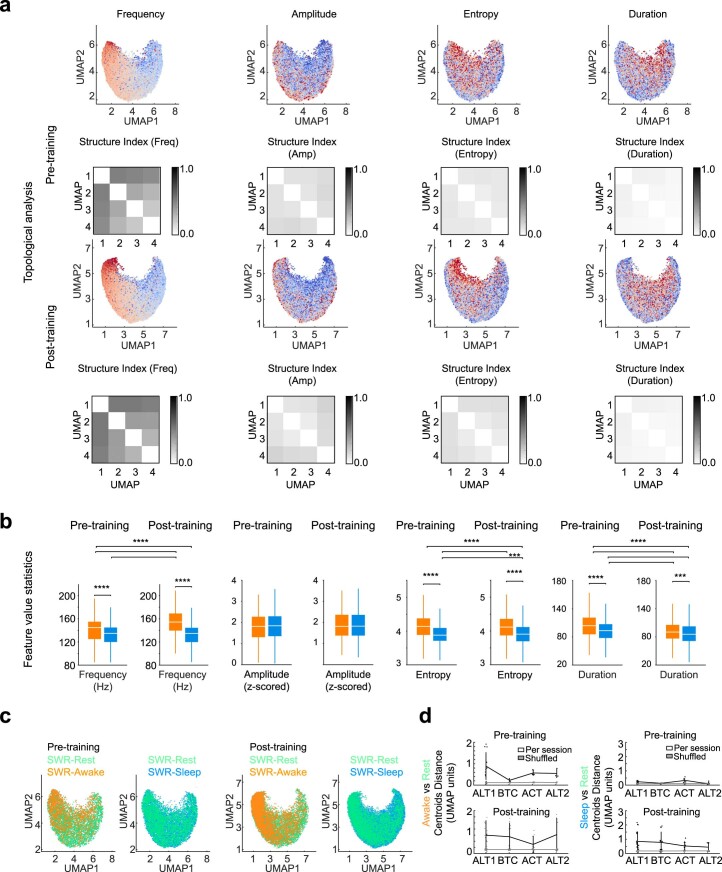
Analysis of SWR across states and conditions. **a**, UMAP1,2 and structure index matrices per feature in the different projections before and after training. **b**, Median features values from all SWR recorded Pre (n = 2706 Awake and n = 9259 Sleep) and Post-training (n = 6458 Awake and n = 29890 Sleep), shown as horizontal bars in the box plots, with the first and third quartiles as box limits. Whiskers indicate the data point further from quartile values that is within 1.5 times the interquartile range. Statistics analysis with a two-way ANOVA for Frequency: Awake/Sleep F(1,1) = 433, p < 0.00001; Pre/Post F(1,1) = 1862, p < 0.00001 and interaction p < 0.0001. Entropy: Pre/Post F(1,1) = 3071, p < 0.00001; interaction p = 0.0023; Duration: Awake/Sleep F(1,1) = 893, p < 0.0001; Pre/Post F(1,1) = 567, p < 0.00001 and interaction p < 0.0001. Post-hoc tests with Tukey-Kramer two-tailed tests ***, p < 0.001; ****, p < 0.0001. **c**, UMAP embedding pre- and post-training showing distribution of Rest SWR as compared to Awake and Sleep events. **d**, Mean distance between centroids for the embedding distribution of Awake versus Rest (left) and Sleep versus Rest (right) SWR recorded Pre- and Post-training across tasks. Plots reflect the mean ± SD centroid distance for all possible combinations of sessions and the three UMAP1 projections. Awake vs Rest (Pre: n = 12 combinations for ALT1, n = 3 for BTC, n = 6 for ACT and n = 3 for ALT2; Post: n = 24 for ALT1, n = 12 for BTC, n = 9 for ACT and n = 9 for ALT2). Sleep vs Rest (Pre: n = 9 combinations for ALT1, n = 3 for BTC, n = 6 for ACT and n = 6 for ALT2; Post: n = 21 for ALT1, n = 12 for BTC, n = 9 for ACT and n = 9 for ALT2). Data was bootstrapped (black), and tested against the shuffled distribution (100 shuffles, gray). No effect of tasks (one-way ANOVA, p > 0.05).

Similarly to data above, SWRs exhibited more SI for frequency in both the original and the reduced space, built separately for events recorded before and after the tasks (Fig. 6b; intrinsic dimension of 4 in all cases). This observation cannot be explained by differences between sessions (Extended Data Fig. 8d), nor by the different rate of SWRs (Extended Data Fig. 8e; bootstrapped). Visualization of SWR features projected over the reduced embedding confirmed these trends (Fig. 6c and Extended Data Fig. 9a). Note that while the embedding is rotated as compared with that from head-fixed recordings, the relationship between the distributed features is preserved due to UMAP invariance.

We first focused on evaluating the influence of brain state (awake/rest/sleep) on the organization of SWRs. Analysis of the distribution of awake SWRs revealed remarkable biases, especially in the post-training embedding (Fig. 6c), which were dominant along some UMAP projections (that is, UMAP1 versus UMAP2/3/4 projections; Fig. 6d). We next compared the effects of awake and sleep states before and after tasks to evaluate their potential mechanisms. We estimated the topological distance between the centroids of awake and sleep SWRs per UMAP projection, and confirmed a major influence of training in their separation (Fig. 6e). Standard statistical comparison of awake and sleep events provided only a partial view (Extended Data Fig. 9b).

To dissect these effects closely, we bootstrapped all SWRs for each task/session in the UMAP1 versus UMAP2/3/4 projections, and tested them against the shuffled distribution. We found that the nature of the performed tasks had a major influence on segregating awake and sleep SWRs recorded after training but not before (Fig. 6f). Novelty (tasks ALT1 and BTC) and new learning (task ALT1) had major impact, as reflected in larger centroid separation between post-training awake and sleep SWRs (Fig. 6f). This maximal segregation of SWRs from the first session was consistent for all animals, and may reflect the major role of hippocampus in one-shot learning. Instead, centroid separation decreased significantly for repeated contexts (ACT) and task (ALT2). These results suggest that awake SWRs become more similar to sleep and rest SWRs after habituation to tasks. Instead, SWRs during sleep and rest distributed more homogeneously (Extended Data Fig. 9c) with no effects across tasks (Extended Data Fig. 9d).

**Extended Data Fig. 10:**
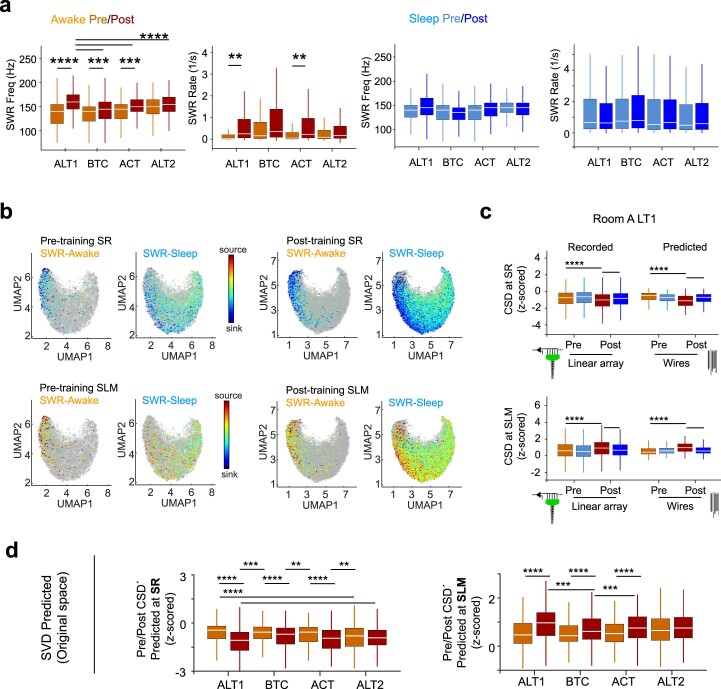
Topological analysis of Pre/Post SWR. **a**, Median frequency and rate of Pre/Post SWR recorded during Awake (left) and Sleep conditions. Only mice recorded with wires and tested in all tasks were included in the analysis (6 mice). Box plots at left show the median Pre/Post SWR frequency and rate during Awake condition per task, as horizontal lines (n = 510/1834 events from Pre/Post in ALT1; n = 821/1692 events from Pre/Post in BTC; n = 417/1007 events from Pre/Post in ACT; and n = 425/834 events from Pre/Post in ALT2). Box plots at right show the median Pre/Post SWR frequency and rate during Sleep condition per task, as horizontal lines (n = 1824/2700 events from Pre/Post in ALT1; n = 1972/8706 events from Pre/Post in BTC; n = 555/5227 events from Pre/Post in ACT; and n = 1774/4599 events from Pre/Post in ALT2). Significant effects in a two-way ANOVA for Awake SWR frequency (state: F(3,1) = 132.3, p < 0.0001; task: F(3,1) = 48.7, p < 0.001) and rate (state only F(3,1) = 27.0, p < 0.0001). Asterisks indicate significant differences of post-hoc Tukey-Kramer two-tailed tests: **, p < 0.01; ***, p < 0.001; ****, p < 0.0001. No significant effects for Sleep SWR. **b**, CSD values recorded at SR and SLM using linear array silicon probes. Data shown for Awake and Sleep SWR as projected in the pre- and post-training UMAP1,2 projection. **c**, Comparison between Pre/Post and Awake/Sleep CSD signals of SWR recorded with the linear arrays versus those predicted from wire recordings in Room A LT1. Box plots show the median CSD value (horizontal lines) from all recorded SWR (SR: n = 559 Pre-Awake, n = 1630 Pre-Sleep, n = 1011 Post-Awake and n = 7870 Post-Sleep; SLM: n = 474 Pre-Awake, n = 1168 Pre-Sleep, n = 829 Post-Awake and n = 7137 Post-Sleep) and predicted SWR in the reduced space (n = 491 Pre-Awake, n = 1602 Pre-Sleep, n = 1693 Post-Awake and n = 2405 Post-Sleep; same for SR and SLM). Box limits indicate the first and third quartiles. Whiskers indicate the data point further from quartile values that is within 1.5 times the interquartile range. **d**, Pre/Post CSD’ values predicted at SR and SLM from wire recordings using the SVD trained in the original space using data from linear arrays. Box plots show the median CSD’ value (horizontal lines) from all predicted SWR per task (n = 491/1693 Pre/Post for ALT1, n = 764/1531 Pre/Post for BTC, n = 398/918 Pre/Post for ACT, n = 395/781 Pre/Post for ALT2). Box limits indicate the first and third quartiles. Whiskers indicate the data point further from quartile values that is within 1.5 times the interquartile range. Significant differences across tasks in Awake SWR (ANOVA F(1,7) = 45, p < 0.0001 at SR; ANOVA F(1,7) = 54, p < 0.0001 at SLM). Post-hoc tests with Tukey-Kramer two-tailed tests **, p < 0.01, ***, p < 0.001; ****, p < 0.0001.

To focus on the cognitive effects, and to exclude potential differences between embedded data, we evaluated their distribution across tasks by building the high-dimensional and low-dimensional representations of events recorded before and after the tasks together, for the awake and sleep conditions separately (Fig. 6g and Extended Data Fig. 10a). The distribution of bootstrapped awake SWRs before and after the tasks exhibited maximal separation in different rooms (novelty; ALT1 and BTC) and for the first track (original training; ALT1), and dropped to shuffle distribution with habituation (experience; ALT2; Fig. 6h). Instead, pre/post SWRs recorded during sleep distributed homogeneously and did not differ from shuffled data (Fig. 6h).

### Topological decoding of inputs underlying ripple variability

**Fig. 7:**
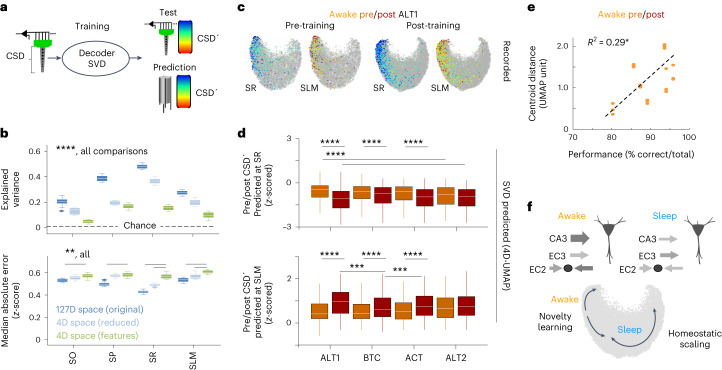
Topological decoding of input mechanisms underlying pre/post differences. **a**, Linear array recordings from the first task (ALT1) were used for training an SVD model to predict CSD values from the waveform space. Once validated, the decoder was applied to wire recordings to evaluate changes across tasks. **b**, Explained variance of CSD values from the linear array test set (*n* = 10 tests), as predicted by the SVD (chance level of shuffled data is shown). Box plots show the median explained variance (horizontal line) at all layers as estimated from the tenfold predictions (*n* = 10 tests), with the first and third quartiles as box limits. Whiskers indicate the data point farthest from quartile values that is within 1.5 times the interquartile range. Two-way ANOVA effects for layer (*F*(3,2) = 487), spatial dimension (*F*(3,2) = 987) and interaction (*F*(3,2) = 53) all at ***P < 0.001 and ****P < 0.0001 for all post hoc comparisons with a Tukey–Kramer two-tailed test. The median absolute error exhibited differences for the interaction between methods and layers (*F*(3,2) = 5.6; *P* < 0.0001). **P < 0.01 for post hoc as indicated. **c**, Pre/post CSD values recorded during ALT1 using linear arrays as projected in the 4D UMAP embedding. **d**, Pre/post CSD′ values predicted at the SR and the SLM from wire recordings using the SVD trained in the 4D UMAP space with linear array data. Box plots show the median predicted CSD′ per task (horizontal line) for all pre/post SWRs as input data points (*n* = 491/1,693 for ALT1 pre/post, *n* = 764/1,531 for BTC pre/post, *n* = 398/918 for ACT pre/post and *n* = 395/781 for ALT2 pre/post; *n* = 4 independent sessions, 6 mice). Box limits indicate the first and third quartiles. Whiskers indicate the data point farthest from quartile values that is within 1.5 times the interquartile range. Significant differences across tasks (two-way ANOVA *F*(1,7) = 58, *P* < 0.0001 at the SR; ANOVA *F*(1,7) = 54, *P* < 0.0001 at the SLM). **P < 0.01 and ****P < 0.0001 for all post hoc comparisons with a Tukey–Kramer two-tailed test. **e**, Correlation between performance in the ALT1 alternate task and the centroid separation in UMAP1 versus UMAP2/3/4 projections between pre/post awake SWRs. Data are from three UMAP projections per mouse (*n* = 7 mice with pre/post sessions with >200 SWR). *P < 0.05. **f**, Summary of the findings from topological analysis of SWR.

Finally, we took advantage of topological decoding and used mice with linear array recordings from the first day (ALT1; Extended Data Fig. 10b) to train and test an SVD model (Fig. 7a). Using these data, we found that CSD values reconstructed from the high-dimensional space were more accurate than those obtained from the reduced UMAP embedding (Fig. 7b, top; *P* < 0.0001; two-way analysis of variance (ANOVA) for methods and layers), suggesting that low-dimensional representations may even lose some information when large cognitive load is at play. Strikingly, the explained variance of layer-specific CSD values from freely moving recordings was similar to that from head-fixed data in the original space (nonsignificant two-way ANOVA), while predicted CSD values from the 4D feature space yielded even poorer results closer to chance level at zero (Fig. 7b). Notably, prediction errors from the SVD trained in the low-dimensional and high-dimensional spaces were rather similar (Fig. 7b).

To ease visualization across tasks, we next sought to apply the SVD trained in the 4D embedding while tracking results in the original space. We found that CSD′ values predicted from wires were roughly similar to CSD values recorded with the linear arrays in the same ALT1 task (Fig. 7c and Extended Data Fig. 10c). We then estimated CSD values at the SR and the SLM of pre/post awake SWRs from wire recordings across tasks using the SVD trained in the 4D UMAP space, and found significant differences (Fig. 7d; see Extended Data Fig. 10d for SVD in the original space). These results support the idea that changes of awake SWR distribution result from different input pathway activity induced after learning. Consistently, the centroid distance between pre/post awake SWRs estimated in the dominant UMAP projections significantly correlated with alternation performance in the ALT1 task (*R*^2^ = 0.29, *P* = 0.0115, Fig. 7e; no correlation with speed or total distance), consistent with major roles of awake SWRs in signaling novel experience and learning.

## Discussion

Using topological and low-dimensional analysis of ripple waveforms recorded within the CA1 cell body layer, we demonstrate that their variability can be precisely quantified and mechanistically explained. We found that SWRs distribute along a continuum of waveforms, which reflect layer-resolved information. For decades, observation of the effect of brain state and cognitive demands on SWRs has remained elusive with changes in frequency, rate, amplitude and the content of replay being described. Here, we show that the intricacy of the accompanying changes can only be partially extracted using statistical and spectral methods. Instead, transforming classification of ripple waveforms into a topological problem reveals dominant mechanistic biases of input pathways.

Uncovering the diversity of SWRs is key to understanding their roles in memory function and dysfunction^1,12^. The attempts to categorize these events based on discrete clusters have been typically confronted with difficulties in defining clear-cut entities^7,10,18–20^. Our topological analysis provides support to the idea that the SWR waveforms represent a continuum, which can be embedded into a low-dimensional space. Similar strategies can be applied to the study of other types of oscillations and LFP signals^33,34^. This permits visualization of the distribution of predefined features such as frequency and amplitude, which can be quantified at the original and the reduced spaces using informational geometry^23,35^. While different SWR categories can be defined using clustering strategies, their interpretability and relationship with specific ripple waveforms will not be necessarily obvious. Future work can examine the relationship between the continuity of ripple waveforms and their categorization from local ensemble patterns and large-scale brain dynamics^7,10,19,20^.

Our analysis provides mechanistic support for interpreting changes of ripple waveforms associated with brain states and learning. Instead of relying on abstractly reduced representations, we chose to evaluate the intrinsic dimension of the data cloud for constraining analysis and visualization. The distribution of SWR waveforms carried layered information on the associated input pathways, which can be extracted from the topological organization in both the original and the reduced space. A decoder trained in both representational spaces successfully connects individual SWRs with the expected sink-source values without relying on laminar information. This permits inference of the underlying inputs and makes the method interpretable in physiological terms. Importantly, while for inputs arriving at the SR (that is, CA3) the decoder is able to explain more than 60% of the variance, there is more information in the ripple waveforms than can be extracted from input generators alone. In contrast, the variance of CSD values at the SP (mostly reflecting passive currents intermixed with perisomatic GABAergic inputs) is less well explained by the decoding strategy. This is consistent with the idea of a major contribution of the local microcircuit in shaping CA1 dynamics^6,12,17,36,37^ and the very nature of SWR events, which reflect ensemble representations brought about by different input assortments^38,39^. Similarly, other input pathways (for example, thalamic head-directional inputs) can contribute differently to shape SWR waveforms across different recording conditions^40,41^. Therefore, further studies should address what the additional contributions of local cell-type-specific and extra-hippocampal microcircuits are to the variation of SWR waveforms.

We found striking differences between awake and sleep SWRs, consistent with previous results^5^. In contrast with standard statistical methods, our approach allows for characterizing the topological direction of changes, providing physiological explanations. SWR events during exploratory pauses shifted toward the high-frequency and high-amplitude regions of the embedding, with cognitive demands associated to novelty, learning and habituation having major impact in their low-dimensional reorganization (Fig. 7f). Our optogenetically informed analysis suggests that low-amplitude slower (80–100 Hz) and high-amplitude faster (120–150 Hz) ripples might involve CA3 and CA2 inputs distinctly. Consistently, novelty signals characteristic of alertness, which tend to upregulate CA3 activity in novel contexts^42,43^, provide support for the drift of awake SWRs toward the region of the low-dimensional embedding characterized by stronger SR sinks. Similarly, awake SWRs are known to reactivate prefrontal cortical circuits more strongly than during sleep phases^44^ suggesting that their topological segregation can also reflect changes in the strength of cortico–hippocampal interaction^10,20^.

Quite contrastingly, during sleep and prolonged immobility, SWR features fluctuate homogeneously along the embedding, consistent with a homeostatic regularization of brain-wide excitability^45^ (Fig. 7f). The homogeneous topological distribution of sleep SWRs recorded before and after experience likely reflects the large representational variability accompanying memory consolidation^46,47^. During this period, memory traces resulting from experience are synaptically scaled and integrated into existing representations^4,48^. We hypothesize that a diversity of SWRs spanning all along the topological space may be reflecting the myriad of ensembles in the process of consolidation.

Our method allows exploitation of the topological organization of ripple waveforms in the high-dimensional and low-dimensional spaces to inform data-driven analysis. Here, we projected well-known features such as the frequency, amplitude and CSD values of SWRs to illustrate how information can be inferred from the data cloud. However, the low structural values of some of these features suggest additional mechanisms may be required to fully explain waveform variability, such as the local cell-type-specific microcircuits and other input pathways mentioned above. By projecting the firing rate from different cell types from the local circuit and afferent regions, our method can help to inform on their different contribution. Finally, topological analysis of SWR waveforms can facilitate identification of the mechanisms underlying disease-specific alterations, such as fast ripples in temporal lobe epilepsy^7^, or slow-frequency ripples in Alzheimer’s disease^49^ and in some forms of interneuropathies^50^. Such a level of understanding of SWR variability using topological and low-dimensional analysis provides a unique opportunity to better dissect the microcircuit mechanisms underlying hippocampal memory function and dysfunction.

## Methods

### Animals

Male and female mice (*Mus musculus*) between 2 and 12 months of age were used in this study. All protocols and procedures were performed according to the Spanish legislation (R.D. 1201/2005 and L.32/2007) and the European Communities Council Directive 2003 (2003/65/CE). Experiments were approved by the Ethics Committee of the Instituto Cajal, the Spanish Research Council (CSIC) and Comunidad de Madrid (protocol no. PROEX 162/19). Experiments included in this paper follow the principle of reduction, to minimize the number of animals. Thus, we obtained several sessions (electrode penetration) per animal, which were treated as independent observations. Whenever critical for the scientific question at hand, data are reported by animals. Mice were housed either alone or together with others to secure their well-being (for example, when implants were compromised and/or there was a dominant mouse in the cage requiring separation). They were maintained in a 12-h light–dark cycle (07:00 to 19:00) at 21–23 °C and 50–65% humidity with access to food and drink ad libitum.

### Study design

Mice from different lines were randomly assigned to head-fixed and freely moving experiments, as described below. No statistical method was used to predetermine sample sizes, which were similar to those reported previously for this type of study^7,29^. Data collection was not performed blind to the conditions of the experiments (that is, head-fixed, freely moving, optogenetic stimulation, sleep, awake, tasks) due to execution requirement. For data analysis, detection of SWRs was blind to the topological analysis. All SWR events and recording sessions were used, except for analysis requiring specific inclusion criteria (for example, sleep, rest, awake conditions), which are indicated in the corresponding section.

#### Head-fixed electrophysiological recordings from awake mice

Adult wild-type C57BL/6 male and female mice were implanted with fixation head bars under isoflurane anesthesia (1.5–2% mixed in oxygen; 400 ml min^−1^). Two silver wires, previously chlorinated, and screws were inserted over the cerebellum for reference/ground connections. Implants of wires and screws were secured to the skull with light-cured glue (Optibond Universal, Kerr Dental) and secured with dental cement (Unifast LC, GC America). For optogenetic experiments, mice were injected in the same surgical act with adeno-associated viruses (AAVs) to drive expression at specific hippocampal regions, including: (a) CA2 pyramidal cells, which were targeted by injecting AAV5-DIO-EF1a-hChR2-mCherry (1 µl, 3.4 × 10^12^ viral genomes per ml) in Amigo2-Cre mice^51^ (now available at The Jackson Laboratory, as Amigo2-cre1Sieg/J, 030215); and (b) CA3 pyramidal cells, which were targeted with PHPeB-CamKII-ChRger2-TS-EYFP-WPRE^52^ (0.5 µl, 2.6 × 10^13^ viral genomes per ml at 1:4 dilution in saline) in C57BL/6 mice. Injection coordinates were: for CA2, −1.6 mm anteroposterior and 1.5 mm mediolateral from bregma, depth 1.1 mm; for CA3, −2 mm anteroposterior and 1.75 mediolateral (angle 10°) from bregma, depth 1.9 mm.

All mice were habituated to the head-fixed setup, consisting of a wheel (20 cm radius) coupled to a stereotactic frame. Habituation sessions (5–7 d, two sessions per day) included handling and placing/removing mice on the apparatus for increasing periods of time (from 5–10 min to more than 1 h). Once mice were habituated to staying in the wheel for long periods, a cranial window was opened over CA1 at 2 mm posterior from bregma and 1.25 mm lateral from the midline in each hemisphere under isoflurane anesthesia. Then, the craniotomy was covered with a low-toxicity silicone elastomer (Kwik-Sil, World Precision Instruments).

Recordings started the day after craniotomy and proceeded over the following 3–4 d. Individual penetrations were considered an independent experimental session, thus providing several sessions per animal. At the end of each recording day, the craniotomy was clean and sealed with Kwik-Sil. Mice returned to their home cage until the next recording day.

For recordings, we used a 16-channel silicon probe comprising a linear array with 100 µm resolution and 703 µm^2^ electrode area (A1x16-5 mm-100-703; Neuronexus). For optogenetic experiments, a 105-µm optical fiber was attached to the probe over the 8th–10th electrode from the bottom. Extracellular signals were pre-amplified (4× gain) and recorded with a 16-channel AC amplifier (100×, Multichannel Systems), and sampled at 20 kHz per channel (Digidata 1440, Molecular Devices). Data were acquired with Axoscope (v11). Silicon probes were inserted up to 400–500 µm below the cell body layer of the CA1 region of the dorsal hippocampus to get a laminar profile, including SO, SP, SR and SLM. Relevant LFP events (ripples, multiunit firing, sharp-waves and theta-gamma oscillations) helped to identify penetration.

#### Optogenetic stimulation

To evoke SWRs optogenetically, we applied 100-ms square pulses of light at 0.2–0.33 Hz with a 532-nm-wavelength laser (MGL-FN-532-300 mW, CNI Optoelectronics Tech) to stimulate axon terminals of CA2 pyramidal neurons (SO) and CA3 pyramidal neurons (SR and SO) in the CA1 region. In both cases, the fiber remained over the alveus. The laser power was adjusted in each experiment to obtain physiological-like oscillations comparable to spontaneous SWRs (CA2, 200–1,000 µW; CA3, 2–500 µW). Note we did not use the 473-nm-wavelength light (optimal wavelength to activate channelrhodopsin) because it evoked large amplitude nonphysiological oscillations. In a subset of experiments, we tested half-sinusoidal pulses of 50–100 ms and found similar types of events as generated with square pulses.

#### Electrophysiological recordings from freely moving mice

Adult male and female mice, either wild-type (C57BL/6, in-house) or from the B6.Cg-Tg(Thy1-CO P4/EYFP)18Gfng/J (JAX mice, 007612) line, were implanted with optrodes consisting of four tungsten wires (0.002-inch, bare; 0.004-inch, PFA coated; AM Systems) coupled to optic fibers (200 µm diameter; Thorlabs). The wire tips protruded between 100 µm and 400 µm from the fiber flat surface (located at the alveus) allowing for laminar recordings around the SP and the SR. Implants targeted both hemispheres (anteroposterior: −2.5 mm; mediolateral 2.2 mm from bregma; −1.1 mm depth from the dura). Once the wires were located in their final position, the shanks were glued to the skull with OptiBond Universal (Kerr Dental, Switzerland) and secured with light-cured acrylic resin (Unifast LC, GC Corporation).

In addition, some adult C57BL/6 wild-type mice were implanted with 32-channel silicon probes (A4X8-5mm 100-200-413; Neuronexus). Animals were anesthetized with isoflurane (2% for induction, 1–2% for maintenance, 500 ml min^−1^) mixed with oxygen. Probes targeted the right dorsal hippocampus (anteroposterior: −2.0 mm; mediolateral: 1.5 mm from bregma; −1.7 mm depth from the dura). Reference and ground electrodes were placed at the skull above the cerebellum. Once in place, silicon probes were covered by Vaseline and cemented to the skull. A grounded copper mesh cage was built to protect the probes and to ground the system. All mice received doses of enrofloxacin (20 mg per kg body weight), dexamethasone (0.2 mg per kg body weight) and buprenorphine (0.05 mg per kg body weight) subcutaneously on the day of surgery and 24 h later.

Animals were allowed to recover for at least a week before habituation began. Signals were recorded at 30 kHz with an Open Ephys system using an Intan RHD2132 32-channel head-stage, including a 3-axis accelerometer (Intan Technologies). Data were acquired with Open Ephys GUI 0.4.6.

#### Freely moving tasks and recordings

The experimental protocol consisted of four tasks done with at least 3 d of separation between them. Behavioral tasks started after a habituation phase consisting of at least 3 d of handling, followed by 2 d of habituation to the recording box. This box, used throughout the experimental protocol, consisted of a black polypropylene enclosure (28 cm × 22 cm × 42 cm height) with bedding up to 2 cm. Habituation took place in the familiar room where mice were about to run the first round of tasks (room A). Animals were water deprived for 24 h before the tasks. Tasks lasted 20 min each, and SWRs were recorded immediately before (pre) and after (post) during 2 h in the home cage. Habituation mimicked the behavioral tasks, and consisted of 2 h of recording (pre), followed by a 15-min exposition to the home cage with access to water, and then back to the recording box for another 2 h of recording (post).

The first behavioral tasks consisted of an alternation task in a linear track (LT1; 74 cm long × 7 cm wide, with 12-cm-tall walls) located in the already familiar room A. This linear track had visual (three vertical white stripes on each side) and somatosensory (three polishing paper stripes on the floor) cues in one-half of the corridor. Mice were transported to the maze and allowed to run for reward (4 µl sugar water, 10%) during 15 min. Rewards were automatically delivered through a water valve, which was activated by an infrared sensor controlled by an Arduino system. A reward was delivered only if mice successfully alternated in the maze.

The second task consisted of free exploration (15 min) of a two-chamber place preference enclosure (TC, chambers of 18 cm × 20 cm and 25 cm height, connected by a 7-cm-wide corridor) located in a room the mice had never visited (room B). This task was the only one that did not require the animals to be water deprived and was used to test for effects of novelty in the absence of training and reward.

For the next two tasks, mice returned to the familiar room A. The third task was run in a semicircular track (CT; 120 cm long × 7 cm wide, 2-cm-tall walls) with somatosensory cues like in the linear track, where animals had to alternate (15 min). Water port rewards were available at both extremes of the semicircular track.

Finally, the fourth task consisted of a repetition of the first linear track (LT2) over 15 min. Mice carrying wires performed all the tasks in a row. Mice carrying silicon probes were recorded only during the first task (LT1) to provide data for CSD analysis.

#### SWR detection and feature analysis

For detecting SWR events, we followed consensus criteria^2^. First, we removed noisy epochs determined by excessive signal similarity between two separated recording channels (for example, masticatory artifacts). LFP signals from these channels were summed, and epochs deviating >10 times the s.d. from the mean were deleted.

Next, we selected the SP channel from the different shanks and/or wires, which was characterized by the larger ripples and MUA firing, as judged from the maximal power in the ripple (100–250 Hz) and MUA bands (300–400 Hz), respectively. SP signals were filtered (forward-backward-zero-phase finite impulse response filter of order 512 implemented in either MATLAB 2020a and 2021b (MathWorks) between 70 Hz and 400 Hz, and the envelope calculated with a fourth-order Savitzky-Golay filter with a window duration of 33.4 ms, followed by two smoothing moving windows of 2.3 and 6.7 ms, using the ‘movmean’ function. We intentionally left the bottom filter cutoff at 70 Hz to allow for detection of a wide diversity of SWR events, including slow SWRs of 80–100 Hz, similar to those recorded in primates. The upper filter at 400 Hz permitted detection of MUA firing, which is typically used for replay studies. These detection limits are within the ranges reported by consensus^2^. Importantly, all candidate SWR events were validated (see below). A MUA index was estimated from the area of the spectral power at 300–400 Hz bandwidth.

For detection, candidate events were detected by thresholding over 2–5 s.d. of the envelope signal. Detected events closer than 15 ms were merged. All candidate events were centered by the minimum value of the waveform closer to the peak of the envelope using a 30-ms window. Finally, an expert validated all candidate events using a custom-made MATLAB GUI. Validation was based on the following criteria: (a) a clear LFP ripple oscillation should be confined to SP, sometimes intermixed with MUA; (b) the ripple should be associated with a sharp-wave at SR. Importantly, all events are detected from non-theta periods.

Analysis of LFP signals was implemented in MATLAB. To estimate SWR features of validated events, raw signals at the SP (ripples) and the SR (sharp-waves) were filtered in different bands. The amplitude of the ripple was defined from the envelope of the 70–400 Hz filtered SP signal. We deliberately chose a wide frequency range to evaluate potential segregation between events in the fast gamma (80–100 Hz) and the ripple (>120 Hz) bands. The slopes were defined for both the ripples and the sharp-waves using a 1–10 Hz filtered signal from the SP and the SR, respectively. Slopes to (slope-to-peak) and from (slope-from-peak) the peak were defined similarly from both signals using a linear fit. These features were estimated in the ±25 ms window centered on the ripple peak.

The ripple spectral features were computed from the individual power spectra of the SP channel. The ripple frequency was defined as the power peak (estimated from the spectral bump) in the 70–400 Hz range. To account for the exponential power decay in higher frequencies, we subtracted a fitted exponential curve (‘fitnlm’ from MATLAB toolbox) before looking for the ripple frequency. The spectral entropy was computed from the normalized power spectrum (divided by the sum of all power values along all frequencies) as:

$$Entropy=-\sum Power(\,f\,)\cdot lo{g}_{2}(Power(\,f\,))$$

Where *f* is the frequency binned at 10 Hz. The spectral entropy has been described as useful for characterizing normal and pathological SWRs^7^. The ripple duration was estimated either directly from the envelope of the 70–400 Hz filtered SP signal or from the AUC of the amplitude-normalized 70–400 Hz filtered SP signal, using extended windows of ±100 ms around the peak. To validate estimation of SWR duration, we manually tagged the onset and end of SWRs using three sessions (259 events).

CSD signals were calculated from the second spatial derivative. We included only those sessions meeting spatial criteria (at least eight channels covering continuously from SO to SLM layers). Smoothing was applied to CSD signals for visualization purposes only. Tissue conductivity was considered isotropic across layers. ICA was applied to dissect the different spatial generators^53^, using the ‘runica’ and ‘icaproj’ functions from the EEGLAB ICA toolbox (https://sccn.ucsd.edu/eeglab/index.php). Each session was analyzed separately, and the ICA initialization matrix was always the identity matrix to reproduce the order of components. After excluding ICA components corresponding to noise and artifacts, the remaining SWR-associated ICA spatial profiles were visually inspected and only those fitting the definition of input current generators were selected (1,789 events). Definitions include: (a) the CA2 SWR generator characterized by a sink at SO and a source at the SP/SR border; (b) the CA3 generator characterized by sinks at SO and SR flanking a source at the SP (contralateral), or those associated to SR sinks and SP sources (ipsilateral); (c) the EC3 generator characterized by a sink at deep SLM layer with a source at SR; and (d) the EC2 di-synaptic inhibition generator characterized by a source at the SLM and a sink at the SR. These definitions were derived from the existing knowledge regarding cell-type-specific input pathways^54,55^.

#### Histological analysis

Upon completion of experiments, all mice were deeply anesthetized with sodium pentobarbital (300 mg per kg body weight) and transcardially perfused with PBS (pH 7.4) followed by 4% paraformaldehyde and 15% saturated picric acid in 0.1 PBS. Brains were post-fixed and cut into 50-μm coronal sections in a vibratome (Leica VT 1000S).

Selected sections were washed in 1% Triton X-100 (Sigma) in PBS (PBS-Tx), treated with 10% FBS in PBS-Tx for 1 h, and incubated overnight with the primary antibody solution: rabbit anti-PCP4 (1:100 dilution; Sigma, HPA005792) in 1% FBS in PBS-Tx. After three washes in PBS-Tx, sections were incubated for 2 h at room temperature with the secondary antibody: donkey anti-rabbit Alexa Fluor 647 (1:200 dilution; Invitrogen, A-32795), in PBS-Tx-1% FBS. Following 10 min of incubation with bisbenzimide H33258 (1:10,000 dilution in PBS; Sigma, B2883) for labeling nuclei, sections were washed and mounted on glass slides in Mowiol (17% polyvinyl alcohol 4–88, 33% glycerin and 2% thimerosal in PBS).

Multichannel fluorescence stacks were acquired in a confocal microscope (Leica SP5), with the LAS AF software v2.6.0 build 7266 (Leica), and objectives HC PL APO CS 10.0 × 0.40 DRY UV or HCX PL APO lambda blue 20.0 × 0.70 IMM UV. The pinhole was set at 1 Airy unit, and the following channel settings were applied (fluorophore, laser, excitation wavelength, emission spectral filter): (a) bisbenzimide, Diode, 405 nm, 415–485 nm; (b) EYFP or track autofluorescence, Argon, 488 nm, 499–535 nm; (c) mCherry, DPSS, 561 nm, 571–620 nm; (d) Alexa Fluor 647, HeNe, 633 nm, 652–738 nm. For epifluorescence imaging, a microscope (LEICA AF 6500/7000) with a 10 × 0.3 dry objective and the following filters were used (excitation, dicroic, emission spectral filters): N2.1 (BP515-560, LP590, 580). Fiji software (National Institutes of Health Image; v.2.13.0) was used for subsequent image adjustment and analysis.

Quantification of mCherry^+^/PCP4^+^ cells were made in ×20 confocal images at one confocal plane per mouse. For illustration purposes, *z*-projections (average intensity) were made. Estimation of CA3 infection was achieved in ×10 epifluorescence images, measuring the linear extension along the pyramidal layer for both the EYFP^+^ region and the complete CA3 region (from CA3c at the hilus to the border with CA2 defined by PCP4). These analyses were made in one or two sections for each animal at around −2 mm anteroposterior from bregma, coinciding with the recordings coordinate.

#### Methods for estimation of the intrinsic dimension of the waveform space

Our topological method starts by projecting the ripple waveforms in a high-dimensional space determined by the temporal sampling rate. To build the high-dimensional space, we first downsampled SP signals to 2,500 Hz and cut ±25-ms windows around the peak of detected and filtered SWRs (rounded to 127 points). Projecting all SWRs into the 127D space (one dimension per sample, one point per SWR) resulted in a data cloud, which could be recovered into a low-dimensional space. This idea was inspired by early work on unbiased classification of SWRs using unsupervised methods^7,10,18^. However, instead of predefining the visualization dimension to 2D, we looked for the minimal number of dimensions that preserves the data structure.

We first compared different methods for estimating intrinsic dimension of the data cloud in the 127D space. To this purpose, we used the R library ‘intrinsicDimension’ in Python (version 1.2.0; https://cran.r-project.org/web/packages/intrinsicDimension/vignettes/intrinsic-dimension-estimation.html). This includes methods such as local expected simplex skewness (ESS Local), dimension estimation via translated poisson distributions (MaxL Local) and local PCA (PCA Local). In addition, we used an ABID method, which does not rely on distances but instead estimates the angle distribution in the vicinity of each point^22^.

To validate the different methods, we built the ground truth from several objects in the high-dimensional space, including 2D plane and Swissroll, and a five-dimensional hyperball using codes from the R library. For building a 2D torus, we adapted the R functions to Python. To generate the objects, *N* points were uniformly distributed along the corresponding surface or volume defined by their parametric equations. They were subsequently embedded in 127 dimensions, with added Gaussian noise (s.d. = 0.01) in all directions of space.

#### Synthetic SWRs

In addition to objects, we also simulated synthetic SWRs similarly to experimental events. To generate synthetic ripples, we convolved a sinusoidal signal of a given frequency with a Gaussian signal of a given amplitude and s.d., which defined duration. For each of the three parameters, we used a uniform random distribution of 2,000 samples between the values corresponding to percentiles 5/95% of the real data for the amplitude and the frequency, and between 0.5 and 2 s.d. for duration. Synthetic SWRs were created at the same sampling rate as experimental events. Two different synthetic datasets were built, one with a continuous distribution of frequencies (80–240 Hz); and the other built from three different frequency ranges (80–100 Hz, 130–150 Hz, 190–210 Hz). To make them comparable to experimental SWRs, noise equivalent to the root mean square error of LFP signals was added.

#### Persistent homology analysis

We evaluated the topology of the data cloud directly in the high-dimensional space (127D) using the persistent homology package Ripser.py (https://github.com/scikit-tda/ripser.py/). Persistent homology looks for the persistence of *n*-dimensional simplicial complexes as varying the radius around each data point. The different homology groups are defined from the number of cuts that separate data in pieces of different dimensions (H_0_, H_1_ and H_2_), with the Betti numbers representing the rank of the homology group. In H_0_, the number of connected components that persist after increasing the radius is shown. H_1_ quantifies the number of loops. H_2_ identifies the number of cavities in the data. To validate analysis, we used objects of known topology (torus, ball, plane, and so on) and synthetic SWR data (continuous and 3-clustered distributions). For this analysis, we excluded outliers as in ref. ^56^. Analysis was executed in the supercomputer cluster Artemisa (https://artemisa.ific.uv.es/web/content/nvidia-tesla-volta-v100-sxm2/) using >400 Gb RAM. To this purpose, data were bootstrapped 100 times in groups of 3,500 points and results were tested for consistency across different realizations.

#### Dimensionality reduction techniques

To reduce dimension from the original 127D space to the intrinsic dimension, we used different methods. Isomap was applied using the Python library sklearn.manifold version 0.24.2 (https://scikit-learn.org/stable/modules/manifold.html). We used the UMAP version 0.5.1 (https://umap-learn.readthedocs.io/en/latest/) in Python 3.8.10 Anaconda, which is known to properly preserve local and global distances while embedding data in a lower-dimensional space. A standard PCA was also applied. We found UMAP to be very efficient in computational terms with execution time independent of the number of data points. In contrast, Isomap was computationally costly especially for >10,000 data points. We also tested *t*-SNE^57^, which had a bit better computer efficiency than Isomap, but can reduce space only up to 3D. In all cases, we used default values for reconstruction parameters. Algorithms were initialized randomly. We found UMAP to provide robust results independent of initialization. Because the symmetric Laplacian of the graph G is a discrete approximation of the Laplace Beltrami operator of the manifold, the method uses a spectral layout to initialize the embedding. This provides convergence and stability within the algorithm.

#### Feature space

To evaluate the advantage of UMAP versus simpler approaches, we constructed a space using the SWR features (frequency, amplitude, entropy and duration). In this 4D space, SWRs will form a point cloud similarly to the waveform space, but they will differ in location in the space coordinates and hence their shapes will be different. Note that that neighbors in the 4D feature space will not necessarily be neighbors in the 4D UMAP space.

#### Structure index

We used the SI to quantify the amount of structure the projection of a given feature presents over the data cloud^23^. We started with a data cloud in which each point has a value of an arbitrary feature. First, we divided the feature values into ten equal bins, and then we assigned each point to a group associated with a feature bin (bin group). Next, we computed the pairwise overlap between bin groups as follows. Given two bin groups, ${\mathscr{U}}$ and ${\mathcal{V}}$, we define the overlap score (OS) from ${\mathscr{U}}$ to ${\mathcal{V}}({\mathrm{OS}}_{{\mathscr{U}}\to {\mathcal{V}}})$ as the ratio of *k*-nearest neighbors of all the points of ${\mathscr{U}}$ that belong to ${\mathcal{V}}$ in the point cloud space. That is,

$${\mathrm{OS}}_{{\mathscr{U}}\to {\mathcal{V}}}\left({k}\right)=\frac{1}{{\mathscr{U}}\times k}\sum _{u\in {\mathscr{U}}}{\rm{|}}\Big\{{N}_{u}^{\,j}\left({\mathscr{U}}\cup {\mathcal{V}}-\left\{u\right\}\right){\rm{|}}\;j=1,\ldots ,k\Big\}\cap {\mathcal{V}}$$

where ${N}_{u}^{\,j}\left({\mathscr{U}}\cup {\mathcal{V}}-\{u\}\right)$ is the *j*_th_ nearest neighbor of point *u* in the set ${\mathscr{U}}\cup {\mathcal{V}}-\{u\}$.

Computing the OS for each pair of bin groups (${{\mathscr{U}}}_{a}$ and ${{\mathcal{V}}}_{b}$) yields an adjacency matrix (*A*_*nxn*_) whose entry (*a*,*b*) equals fg. *A* can be thought of as representing a weighted directed graph, where each node is a bin group, and the edges represent the overlap (or connection) between them. We do not allow any self-edges in the weighted directed graph so that we set ${\mathrm{O{S}}}_{{\mathscr{U}}\to {\mathscr{U}}}\left({\rm{k}}\right)=0$.

Finally, we define the SI as 1 minus the mean weighted out-degree of the nodes after scaling it:

$${\mathrm{SI}}\left({\mathscr{M}}\right)=1-\left(\frac{2}{{n}^{2}-n}\,\mathop{\sum }\limits_{a}^{n}\mathop{\sum }\limits_{b}^{n}{A}_{a,b}\right)$$

The SI takes values between 0 (random feature distribution, fully connected graph) and 1 (maximally separated feature distribution, non-connected graph). According to this definition, on small datasets and using a small number of neighbors (*k*), the non-symmetry of *k*-nearest neighborhoods can yield slightly negative values. Thus, we define the final SI to be the maximum of 0 and the result of the equation above. Importantly, by definition the SI agnostic to the type of structure (for example, gradient and patchy). Instead, it is the weighted directed graph that provides additional insights. Note that this metric can be applied to *n*-dimensional spaces and any arbitrary cloud distribution (for example, torus, Swissrolls and planes).

Importantly, for quantitative comparison of structural indices from different features, the same set of points should be used. For instance, since CSD values are typically estimated from a subset of recordings meeting methodological criteria, their structural values cannot be directly compared with that of frequency or amplitude for the full dataset.

#### Spatial correlation analysis

Spatial correlation analysis of SWR features was implemented at 4D by using voxels of different resolutions. To validate the voxel size, a toy model of anticorrelated and random feature distributions was simulated over the 4D experimental SWR embedding. The number of experimental data points per voxels of different sizes (in UMAP coordinates), as well as mean values per feature, were estimated to match the expected correlation of the toy model. The spatial correlation coefficient was calculated using the Pearson correlation between mean voxel features for both the anticorrelated (expected *R*^2^ = 1) and random (expected *R*^2^ = 0) distributions. The optimal voxel size was defined as the value that best optimized the expected correlation for both distributions at 4D (voxel size of 1 corresponding to about 200 events). Note that this is a linear correlation between two features in 4D voxels, not requiring corrections for multiple dimensions.

#### Topological categorization of SWRs in the UMAP embedding

We defined different categories of SWR events in the UMAP embedding by looking at the complementary distribution of different features using Python (3.8.10 Anaconda) with libraries Numpy (1.18.5), SciPy (1.5.4) and Matplotlib (3.3.3). Regions of interest (ROIs) were operationally defined along the topological limits of gradient distribution per feature. To this purpose, we first defined the ranges of interest of the SWR individual features (for example, frequency, amplitude, entropy). For the *n* ripples with feature values in a predetermined range, their coordinates ***X***_*n*_ in the UMAP embedding were used to estimate their probability density $\hat{f}({\boldsymbol{x}})$ in a 2D grid space, ***x***. For this, we computed the bivariate kernel density estimator making use of the seaborn ‘kdeplot’ function with a Gaussian kernel *K* and a smoothing bandwidth *h* determined internally using the Scott method (https://seaborn.pydata.org/generated/seaborn.kdeplot.html). The grid space ***x*** had a size of 200 × 200 points evenly spaced from the extreme values of ${{\boldsymbol{X}}}_{n}$.

$$\hat{f}({\boldsymbol{x}})=\frac{1}{n{h}^{2}}\mathop{\sum }\limits_{i=1}^{n}K\left\{\frac{1}{h}({\boldsymbol{x}}-{{\boldsymbol{X}}}_{i})\right\}$$

The estimator $\hat{f}({\boldsymbol{x}})$ allowed representing the scattered discrete events into a continuous probability density function, which was normalized by the number of ripples *n* such that the total area under all densities sums to 1. Each point of the grid space ***x*** was assigned a density value, which can be considered as a third axis *z*. To visualize the density values as contours in two dimensions, the probability density function was partitioned in 10 levels of the same density proportion in the *z* axis. Each curve shows a level set such that a proportion of the total density lies below it, with contour plots of smallest area representing higher density. The iso-contour that best controlled the over-smoothing and under-smoothing of the distribution was selected for each SWR feature. This was often the 6th or 7th contour from highest to lowest density, which represents 60% to 70% of the highest density iso-proportions. Density contours from each feature were then combined, and the overlapping ROIs were identified.

We also estimated the centroid location of the data cloud by selecting events with different characteristics (for example, percentile values) or SWRs of different origin (for example, sleep/awake; optogenetically evoked, and so on). The distance between centroids or between data points was calculated using the Euclidean distance in UMAP coordinates either in 2D projections or in the reduced 4D space.

For bootstrapping analysis, we subsampled the embedding by picking up a similar number of events for each session/task and repeating this process 10–100 times, resulting in a mean value per session. The sample size was typically 200, 100 or 50 events depending on the analysis and data availability for each observation unit (session). For shuffling, we randomized the SWR coordinates at the UMAP embedding and repeated the process 100 times, resulting in a mean value per session. Bootstrapping and shuffling were performed per UMAP projection and at 4D.

#### Alignment of different datasets

To compare between datasets, we used manifold alignment^58^. To this purpose, the center of mass of points sharing similar bin values of a given feature (20 bins) was estimated for each manifold in the 4D reduced space. The two point sets *{p*_*i*_} and {*p*_*i*_*’*} with *i* = 1, 2,…, 20; follow a one-to-one relation of the form *p*_*i*_’ = *Rp*_*i*_ + *T* + *N*_*i*_, where *R* is a rotation matrix, *T* a translation vector, and *N*_*i*_ a noise vector. Using the algorithm presented by ref. ^58^, we computed the least squares solution of *R* and *T* to calculate the optimal manifold alignment. Once aligned by a given feature, the spatial correlation between features in the two datasets was estimated using the method explained above (UMAP voxels of 1 corresponding to 200 events).

#### Fitting new data into an existing embedding

To align evoked SWRs into an existing embedding, we used spontaneous SWRs of the optogenetic experiments as the control. To avoid on/off effects of light, we used pulses of 100 ms to isolate a ±25 ms window. The window was centered at the power peak of the evoked ripple. Evoked SWRs were aligned into the existing embedding 1 built with the original spontaneous SWRs. To evaluate correspondence, we built a new embedding 2 by pooling together the original events and the spontaneous SWRs from the optogenetic experiments. This provided a reference location for the distribution of both the original and the new spontaneous events in the new resulting embedding 2. In the third step, we used the coordinates of the original events in embedding 1 versus 2 to estimate the error of the original spontaneous events (alignment error) and those fitted (fitting error). Finally, evoked events were aligned directly into the original embedding and their distance distribution was confronted with the fitting and the alignment error of spontaneous events, which were always significantly lower than the data (distance between centroids of CA3-evoked and CA2-evoked SWRs; *P* < 0.00001).

#### Topological decoding of SWR laminar information

To evaluate the explanatory capability of topological representation of SWRs, we adopted a decoder approach to predict laminar information from SWRs (both in the original space and 4D reduced topological spaces, as well as in the 4D feature space). First, we divided the dataset of SWRs with an associated CSD into the training and test sets through a tenfold cross-validation approach. To ensure independence between training and testing in the 4D reduced space, the UMAP embedding was recomputed for each fold using the training set, and then the test set was projected into the fitted space. We then preprocessed the CSD values by dividing each layer by its standard deviation (without subtracting the mean to avoid losing polarity information). Then, a decoder for each CSD layer was trained using the SWR position in the original space, in the 4D reduced space or in the 4D feature space.

To determine the goodness of fit of each decoder, we computed the explained variance regression score between the test CSD values and the predicted ones. To determine a confidence chance level, we evaluated the explained variance of shuffled data. The explained variance was calculated using the following formula:

$${\mathrm{{explained}\,{variance}}}\,\left(\,y,{y}^{{\prime} }\right)=1-\frac{{var}\{\,y-{y}^{{\prime} }\}}{{var}\{\,y\}}$$

where *y* is the original (or the shuffled) variable and *y’* is the predicted variable.

Following this schema, multiple decoders were tested, including Wiener Filter, Wiener Cascade, Extreme Gradient Boosting (XGBoost) and support vector regression, with support vector regression yielding the best performance.

To predict laminar information of SWR without an associated CSD, we input the SWR topological coordinates either in the original space or in the 4D reduced space to all tenfold decoders, and the average CSD prediction was computed. We confirmed that the median error of predictions across layers was roughly at zero level, supporting no bias of the decoder trained either in the original or in the low-dimensional space.

An SV classifier was used by leveraging the sklearn library (C-support vector classification). A tenfold approach was used for training the decoder to classify evoked SWRs from CA3 and CA2 based in their position in the 4D UMAP space. The regularization parameter C was set to 1, and a stationary kernel radial basis function was used as suggested by the library. The accuracy classification score (fraction) was used to evaluate the performance of the trained decoders and tested against shuffling data.

#### Sleep scoring and state classification of SWRs

Brain state scoring was implemented semiautomatically. Information from lateral and ceiling cameras was used to validate movement indices calculated from the head-stage accelerometer. The theta/delta signal was estimated from the time frequency spectrum calculated using the ‘bz_WaveSpec’ function from the Buzcode (https://github.com/buzsakilab/). Periods of immobility were separated from periods of running (awake). Immobility periods were subsequently reclassified as ‘rest’ (no movement awake) and ‘sleep’ based on spectral criteria (skewed distribution of spectral values across time epochs). The maximal power in the 1–35-Hz band was used to identify episodes of REM sleep, which helped to define flanked periods of slow-wave sleep. Sensory thresholds during sleep were tested with mild sound stimulation (clicks), which permitted benchmarking of separate periods of rest and sleep during immobility. All SWRs detected in the different periods were classified accordingly.

#### Standard statistical analysis

Statistical analysis was performed with Python and/or MATLAB. Normality and homoscedasticity were confirmed with the Kolmogorov–Smirnov and Levene’s tests, respectively. The number of replications is specified in the text and figures.

Several-way ANOVAs and/or other non-parametric tests were applied for group analysis. Post hoc comparisons were evaluated with Tukey–Kramer two-tailed tests with appropriate adjustment for multiple comparisons. For two-sample comparisons, the one-tailed and two-tailed Student’s *t*-test or another equivalent test was used. Correlation between variables was evaluated with the Pearson product-moment correlation coefficient, which was tested against 0 (that is, no correlation was the null hypothesis) at *P* < 0.05 (two-sided). In most cases, values were *z*-scored (subtract the mean from each value and divide the result by the s.d.) to make data comparable between experimental sessions and across layers.

### Reporting summary

Further information on research design is available in the Nature Portfolio Reporting Summary linked to this article.

## Online content

Any methods, additional references, Nature Portfolio reporting summaries, source data, extended data, supplementary information, acknowledgements, peer review information; details of author contributions and competing interests; and statements of data and code availability are available at 10.1038/s41593-023-01471-9.

### Supplementary information

Reporting Summary

## Data Availability

Data analyzed in this study are publicly available in Figshare at https://figshare.com/projects/Topological_SWR/125359. This includes ripple waveforms in the 50-ms window (±20 ms) from head-fixed and freely moving experiments, as well as synthetic ripples.

## Code availability

Code used in this study is available in the following interactive notebook: https://colab.research.google.com/drive/1AHG4UQ15NobY2tI7Kc3hQFEkocdRzIsa?usp=share_link#scrollTo=GI8nBd8hOuSv/.

Codes and notebook are also deposited in GitHub at https://github.com/PridaLab/Topological_SWR/.
