# Supplementary material for: Topological analysis of sharp-wave ripple waveforms reveals input mechanisms behind feature variations
Source: Nat Neurosci. 2023 Nov 9;26(12):2171–81. doi: 10.1038/s41593-023-01471-9 (PMC10689241; doi:10.1038/s41593-023-01471-9)
Supplement: Supplementary file 1 — Reporting Summary [file 41593_2023_1471_MOESM1_ESM.pdf]

nature portfolio

Corresponding author(s): Liset M de la Prida

Last updated by author(s): Sep 22, 2023

Reporting Summary

Nature Portfolio wishes to improve the reproducibility of the work that we publish. This form provides structure for consistency and transparency in reporting. For further information on Nature Portfolio policies, see our Editorial Policies and the Editorial Policy Checklist.

Statistics

For all statistical analyses, confirm that the following items are present in the figure legend, table legend, main text, or Methods section.

n/a	Confirmed	
☐	☒	The exact sample size (*n*) for each experimental group/condition, given as a discrete number and unit of measurement
☐	☒	A statement on whether measurements were taken from distinct samples or whether the same sample was measured repeatedly
☐	☒	The statistical test(s) used AND whether they are one- or two-sided<br>*Only common tests should be described solely by name; describe more complex techniques in the Methods section.*
☒	☐	A description of all covariates tested
☒	☐	A description of any assumptions or corrections, such as tests of normality and adjustment for multiple comparisons
☐	☒	A full description of the statistical parameters including central tendency (e.g. means) or other basic estimates (e.g. regression coefficient) AND variation (e.g. standard deviation) or associated estimates of uncertainty (e.g. confidence intervals)
☐	☒	For null hypothesis testing, the test statistic (e.g. *F*, *t*, *r*) with confidence intervals, effect sizes, degrees of freedom and *P* value noted<br>*Give P values as exact values whenever suitable.*
☒	☐	For Bayesian analysis, information on the choice of priors and Markov chain Monte Carlo settings
☒	☐	For hierarchical and complex designs, identification of the appropriate level for tests and full reporting of outcomes
☐	☒	Estimates of effect sizes (e.g. Cohen's *d*, Pearson's *r*), indicating how they were calculated

Our web collection on statistics for biologists contains articles on many of the points above.

Software and code

Policy information about availability of computer code

Data collection	Data acquired with Open Ephys GUI 0.4.6; Axoscope (v11).
Data analysis	External software: Python (3.8.10 Anaconda) with libraries Numpy (1.18.5), SciPy (1.5.4), Matplotlib (3.3.3); R library 'intrinsic dimension' (1.2.0); Isomap library sklearn.manifold (0.24.2); UMAP library (0.5.1); Matlab (v2020a & 2021b). Fiji software (NIH Image; v.2.13.0); Simulations and parameter search was implemented in Artemisa supercomputer infrastructure (https://artemisa.ific.uv.es/web/content/nvidia-tesla-volta-v100-sxm2).<br><br>Codes in this study are available in the following interactive notebook: https://colab.research.google.com/drive/1AHG4UQ15NobY2tI7Kc3hQFEkocdRzIsa?usp=share_link#scrollTo=GI8nBd8hOuSv<br>Codes and notebook are also deposited at Github: https://github.com/PridaLab/Topological_SWR

For manuscripts utilizing custom algorithms or software that are central to the research but not yet described in published literature, software must be made available to editors and reviewers. We strongly encourage code deposition in a community repository (e.g. GitHub). See the Nature Portfolio guidelines for submitting code & software for further information.

Data

Policy information about availability of data

All manuscripts must include a data availability statement. This statement should provide the following information, where applicable:

- Accession codes, unique identifiers, or web links for publicly available datasets
- A description of any restrictions on data availability
- For clinical datasets or third party data, please ensure that the statement adheres to our policy

Data analyzed in this study is available at the public repository: https://figshare.com/projects/Topological_SWR/125359
This includes ripple waveforms in the 50 ms window (±20 ms) from head-fixed and freely moving experiments, as well as synthetic ripples.

Research involving human participants, their data, or biological material

Policy information about studies with human participants or human data. See also policy information about sex, gender (identity/presentation), and sexual orientation and race, ethnicity and racism.

Reporting on sex and gender	Humans were not studied
Reporting on race, ethnicity, or other socially relevant groupings	Humans were not studied
Population characteristics	Humans were not studied
Recruitment	Humans were not studied
Ethics oversight	Humans were not studied

Note that full information on the approval of the study protocol must also be provided in the manuscript.

Field-specific reporting

Please select the one below that is the best fit for your research. If you are not sure, read the appropriate sections before making your selection.

☒ Life sciences ☐ Behavioural & social sciences ☐ Ecological, evolutionary & environmental sciences

For a reference copy of the document with all sections, see nature.com/documents/nr-reporting-summary-flat.pdf

Life sciences study design

All studies must disclose on these points even when the disclosure is negative.

Sample size	No statistical method was used to predetermine sample sizes. All ripples examined in this paper were obtained from multiple recording sessions in a number of mice following the principle of reduction. The total number of ripple events were 10741 events, 58 independent sessions, 27 mice recorded head-fixed; and 59907 events, 36 sessions, 9 mice recorded freely moving. Whenever critical for the scientific question at hand, data is reported by animals. This sample size is similar to those reported before for similar studies (e.g., Valero et al., Neuron 94, 2017 and Grosmark and Buzsaki Sc ience 351, 2016)
Data exclusions	All events and sessions were used. Exclusion criteria were applied to subsets of analysis and indicated in every case.
Replication	The exact number of replications for each experiment is detailed in text and figures (as events, sessions, mice, etc..)
Randomization	Mice from different lines were randomly assigned to head-fixed and freely-moving experiments. Randomization strategies were applied to test distribution against shuffling data (see details for each type of analysis in the Methods)
Blinding	Data collection was not performed blind to the conditions of the experiments (i.e., head-fixed, freely-moving, optogenetic stimulation, sleep, awake, tasks) due to execution requirement. For data analysis, detection of SWR was blind to the topological analysis.

Reporting for specific materials, systems and methods

We require information from authors about some types of materials, experimental systems and methods used in many studies. Here, indicate whether each material, system or method listed is relevant to your study. If you are not sure if a list item applies to your research, read the appropriate section before selecting a response.

Materials & experimental systems

n/a	Involved in the study	
☐	☒	Antibodies
☒	☐	Eukaryotic cell lines
☒	☐	Palaeontology and archaeology
☐	☒	Animals and other organisms
☒	☐	Clinical data
☒	☐	Dual use research of concern
☒	☐	Plants

Methods

n/a	Involved in the study	
☒	☐	ChIP-seq
☒	☐	Flow cytometry
☒	☐	MRI-based neuroimaging

Antibodies

Antibodies used	Primary: rabbit anti-PCP4 (1:100, Sigma HPA005792)<br>Secondary: donkey anti-rabbit Alexa Fluor647 (1:200, Invitrogen, A-32795)
Validation	The PCP4 antibody was validated by the literature and previous testing (Fernandez-Lamo et al Cell reports 2019). The secondary donkey anti-rabbit Alexa Fluor647 antibody was verified by the manufacturer, as declared in their website.

Animals and other research organisms

Policy information about studies involving animals; ARRIVE guidelines recommended for reporting animal research, and Sex and Gender in Research

Laboratory animals	Mus musculus from both sexes and 2-12 months of age. To complain with 3R, in this work we used different mouse lines aimed to target different cell-type specific populations for optogenetic and imaging experiments. This include the following lines: Mouse Amigo2-Cre now avilable as (Amigo2-cre1Sieg/J) Jackson Labs Stock #030215 ; B6.Cg-Tg(Thy1-CO P4/EYFP)18Gfng/J (Jaxmice Stock #007612 and (C57BL/6, in-house (all adult of 2-12months, both sexes)<br>Mice were all housed either alone or together with others to secure their wellbeing (e.g., when implants were compromised and/or there was a dominant mouse in the cage requiring separation). They were maintained in a 12h light–dark cycle (7a.m. to 7p.m.) at 21-23ºC and 50-65 % humidity with access to food and drink ad libitum.
Wild animals	No wild type animals are used
Reporting on sex	Animals from both sex were used
Field-collected samples	NO field collected samples are used
Ethics oversight	All protocols and procedures were performed according to the Spanish legislation (R.D. 1201/2005 and L.32/2007) and the European Communities Council Directive 2003 (2003/65/CE). Experiments were approved by the Ethics Committee of the Instituto Cajal, the Spanish Research Council (CSIC) and Comunidad de Madrid (protocol number PROEX 162/19).

Note that full information on the approval of the study protocol must also be provided in the manuscript.
